# Harnessing engineered laccase and functional carrier materials for efficient dye degradation: From mechanism to application

**DOI:** 10.1186/s40643-026-01148-8

**Published:** 2026-10-29

**Authors:** Yue Zhang, Mengyan Yu, Yuhao Zhang, Chunying Chen, Xueting Yang, Yuchen Kang, Siyu Wu, Yifei Liu, Zhiguo Zhou, Lisheng Qian

**Affiliations:** 1 College of Biomedical and Health Sciences, Anhui Science and Technology University, Chuzhou, 233100 China; 2 College of Food Science and Engineering, Anhui Science and Technology University, Chuzhou, 233100 China

**Keywords:** Dye degradation, Enzyme immobilization, Laccase, Protein engineering, Wastewater treatment

## Abstract

Laccases are copper-containing oxidases that have been widely investigated for the decolorization and degradation of synthetic dyes, including azo, anthraquinone, triphenylmethane, indigo and thiazine dyes. However, their application in dye-containing wastewater remains constrained by enzyme instability under complex effluent conditions, limited oxidation of some recalcitrant or high-redox-potential dyes, mediator dependence, and poor recovery and reuse of free laccase. This review discusses recent progress in laccase-catalyzed dye degradation from a mechanism-to-engineering perspective, linking dye-structure-dependent degradation pathways with laccase discovery, recombinant expression, protein engineering and immobilization strategies. Particular attention is given to comparative evidence across dye classes, fungal and bacterial laccases, engineered variants generated through rational design, directed evolution and semi-rational mutagenesis, and immobilized laccase systems based on nanomaterials, biopolymers, magnetic carriers and porous supports. In addition, the review distinguishes laboratory model dye systems from simulated and real wastewater applications, highlighting the need for more cautious interpretation of decolorization efficiency, degradation evidence and process feasibility. Finally, we outline future prospects for laccase applications in dye degradation, including the mining of novel laccases from extreme environments, artificial intelligence-assisted laccase design, and developing new nano-scale carrier materials, which will be essential for enhancing laccase performance and broadening its application scope.

**Graphical abstract:**

**Figure:**
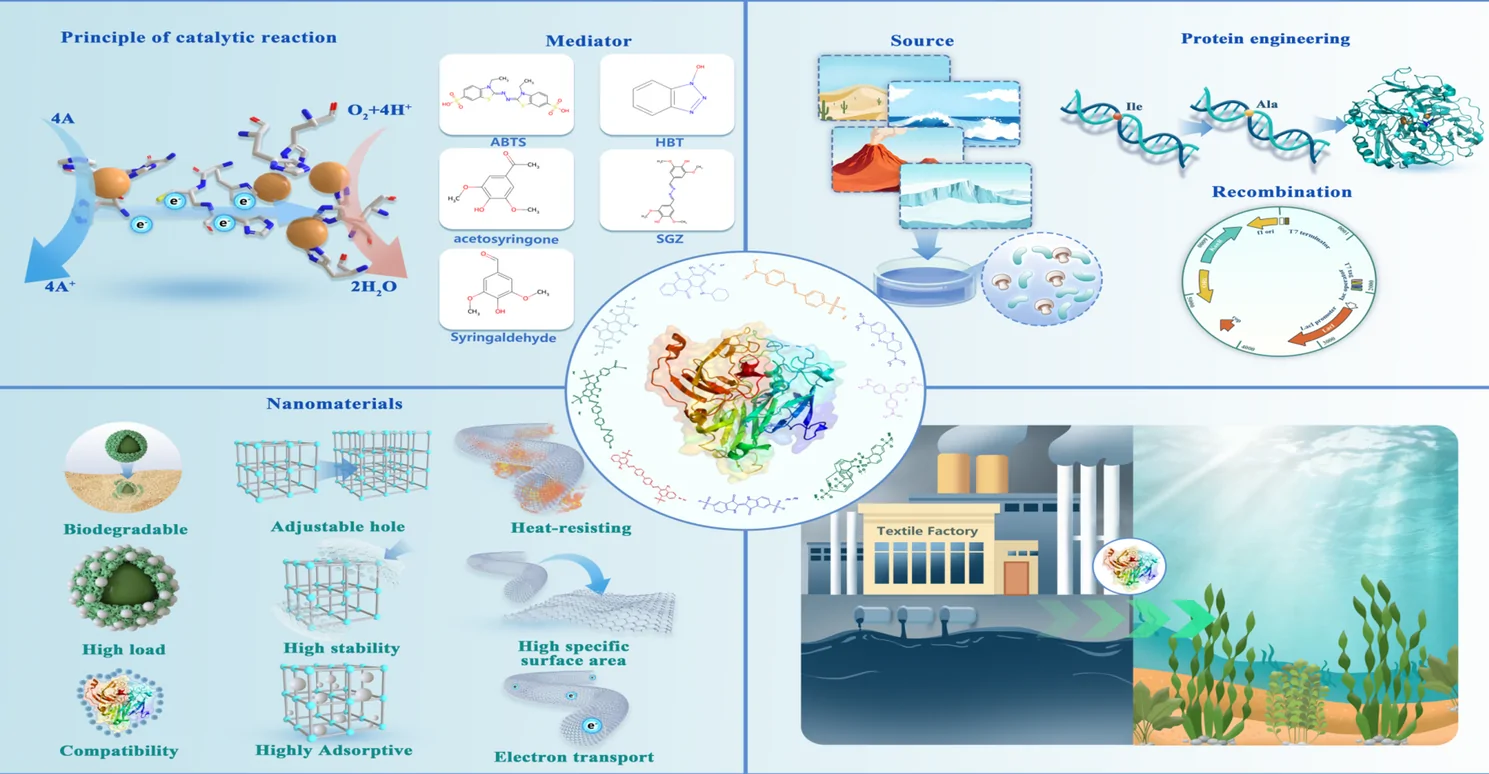

## Highlights

- We review advances in laccase engineering for dye degradation.
- Engineering strategies boost laccase stability, activity, and substrate affinity.
- Functional carriers enhance reusability and adaptability for wastewater treatment.

## Introduction

**Fig. 1:**
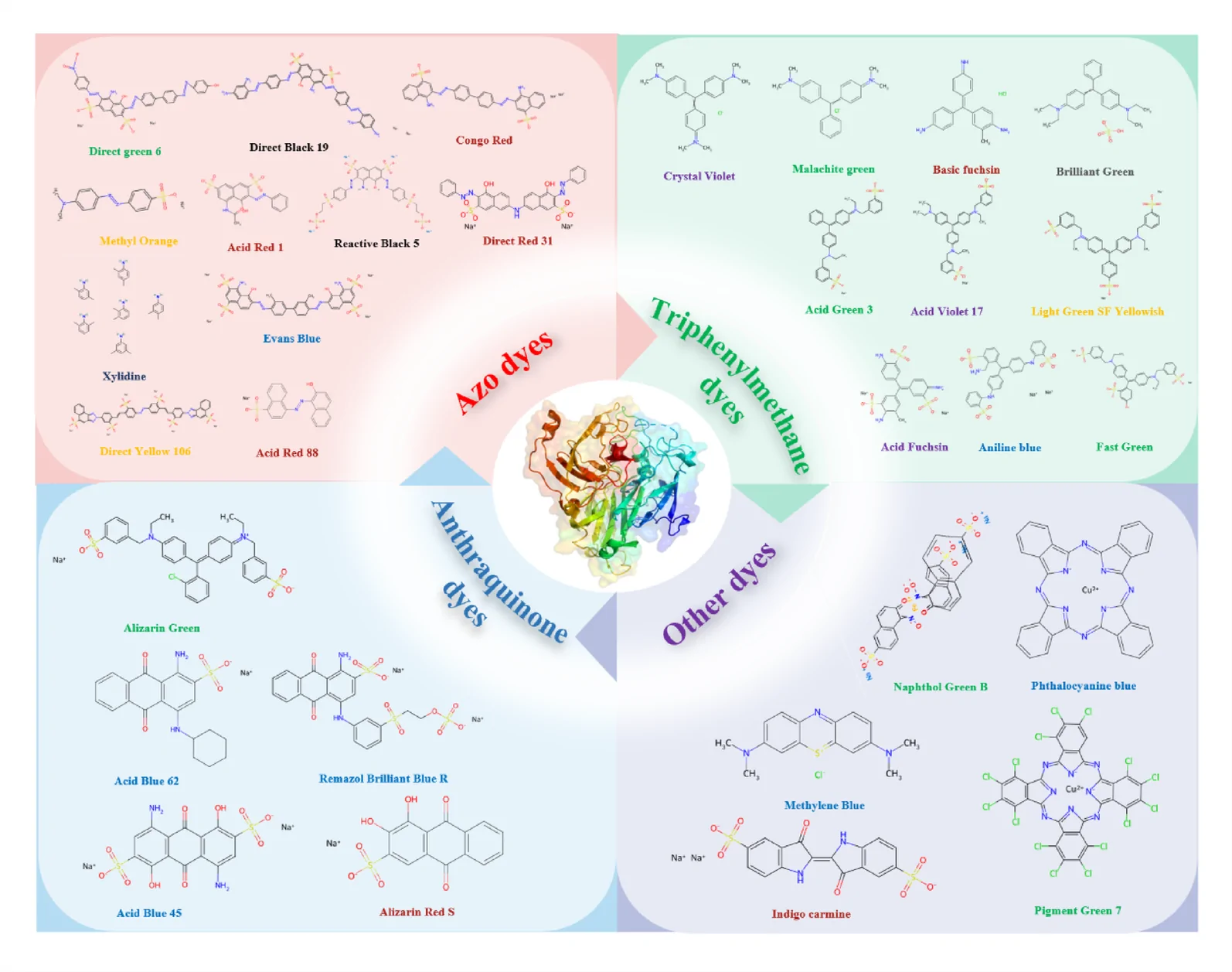
Classes of dyes degraded by laccase

The rising discharge of dye wastewater into natural and human ecosystems poses severe ecological and health hazards (Chen et al. 2021; Singh et al. 2022; Lin et al. 2023). As typical recalcitrant industrial wastewater pollutants, dyes drastically alter pH, dissolved oxygen, temperature, chemical oxygen demand, and salinity levels in receiving waters, and the high salt concentrations in turn inhibit their biodegradation (Zeng et al. 2011; Al-Tohamy et al. 2022). Dye wastewater has carcinogenic and mutagenic properties, primarily due to constituents such as aromatic amines and heavy metals, which are recognised contributors to the increased incidence of bladder and liver cancers and dermatological disorders (Brown and De Vito 1993). Hence, effective dye wastewater treatment is of great significance. Nevertheless, effective dye removal does not necessarily indicate compliance with wastewater discharge standards. Although laccase-based treatment can efficiently reduce color intensity and transform many dye molecules, industrial discharge regulations generally require broader control of chemical oxygen demand (COD), total organic carbon (TOC), toxicity, residual aromatic compounds, salts and other pollutants. Therefore, laccase treatment should be evaluated not only by decolorization efficiency but also by its ability to achieve regulatory requirements or its compatibility with subsequent polishing processes. Importantly, dye decolorization does not necessarily indicate complete degradation or mineralization of dye pollutants. The disappearance of visible color may result from chromophore modification while aromatic intermediates or toxic transformation products remain in the treated effluent. Therefore, reliable evaluation of laccase-based dye treatment requires complementary analytical approaches capable of identifying degradation products, assessing structural transformation, and determining the reduction of residual organic carbon and toxicity. However, dye decolorization remains challenging because of the vast structural diversity of dyes (Fig. 1), encompassing multiple classes such as azo dyes (e.g. Congo Red ([CR]) and Methyl Orange [MO]), Acid Red G, anthraquinones (e.g. Alizarin Red S), and triphenylmethane dyes (e.g. Crystal Violet [CV] and Malachite Green [MG]) (Zhang et al. 2021; Ilić et al. 2026; Zhao et al. 2026). Azo dyes feature the -N = N- bond as a characteristic functional group. Anthraquinones possess an anthraquinone core structure, whereas the triphenylmethane dye core consists of a central carbon atom bonded to three benzene rings (Duxbury 1993; Isaev et al. 2023). Their different core functional groups result in structural specificity, which influences dimensions such as solubility, stability, redox activity, color intensity, and toxicity (Mcyotto et al. 2021).

Compared with conventional physical and chemical treatment technologies, biological methods are promising for dye decolorization because they operate in mild reaction conditions and generate minimal secondary pollution (Methneni et al. 2021; Jain et al. 2022). Conventional physicochemical technologies, including adsorption, coagulation–flocculation, membrane filtration and advanced oxidation processes, have been widely applied for dye wastewater treatment because of their rapid pollutant removal capacity. However, these approaches often involve high energy consumption, chemical requirements, sludge generation, membrane fouling or incomplete mineralization of organic pollutants. In contrast, enzymatic treatment using laccase operates under relatively mild conditions and can selectively transform complex aromatic dye structures with limited secondary pollution. Nevertheless, the lower reaction rate, enzyme instability, operational cost and sensitivity to wastewater composition remain important limitations that must be considered when comparing enzymatic systems with established treatment technologies. Several enzymes participate in dye transformation, including azoreductases, peroxidases and laccases (Wong 1999). Among them, laccase (EC 1.10.3.2) has received sustained attention as a copper-containing oxidase capable of oxidizing a broad range of phenolic and aromatic substrates using molecular oxygen as the terminal electron acceptor (Brugnari et al. 2021; Wang et al. 2025a, b, c). The performance of laccase is governed by both the enzyme and the dye. Fungal laccases often show high redox potential and broad substrate affinity, making them effective against many aromatic dyes (Claus et al. 2002; De Paula et al. 2022; Sodhi et al. 2024). Their application can nevertheless be limited by production efficiency and sensitivity to pH or temperature (Bilal et al. 2019). In contrast, bacterial laccases are highly resistant to high temperatures, acidity, and alkalinity (Strong and Claus 2011; Özdemir et al. 2026). Nevertheless, they exhibit low substrate affinity and are limited in oxidizing substrates with high redox potentials (Imam et al. 2021). Thus, laccase-based dye treatment requires more than identifying an active laccase; it requires matching enzyme properties with dye structure and wastewater conditions.

Recent advances have expanded the possibilities for this matching process. New laccases from fungi and bacteria provide enzymes with diverse catalytic and stability profiles. Heterologous expression improves enzyme availability and facilitates further modification (Rodríguez Couto and Toca Herrera 2006; Mo et al. 2022). The effectiveness of laccase in dye decolorization depends on its redox activity, which is governed by both the structure of the dye and the intrinsic properties of the enzyme. Through rational design, directed evolution, and semi-rational design of amino acid substitutions, laccase can be engineered to fine-tune these inherent characteristics and thus enhance its catalytic efficiency (Khodakarami et al. 2018; Madhavan et al. 2021). The practical application of free laccase is hampered by difficulties in recovery and reuse, leading to high operational costs. Therefore, immobilization strategies, such as adsorption, encapsulation, inclusion, covalent binding, and cross-linking, have gained prominence as they not only facilitate efficient recovery from reaction systems but also significantly bolster enzymatic stability under extreme conditions and against chemical reagents (Zhou et al. 2021; Alvarado-Ramírez et al. 2024). Novel nanomaterials such as graphene, metal-organic frameworks (MOFs), chitosan-based substrates, and β-cyclodextrin offer additional possibilities for enhancing the efficient application of laccase in dye decolorization (Daronch et al. 2020; Hou et al. 2024; Yu et al. 2026). Importantly, enzyme engineering and immobilization engineering should not be considered as independent optimization strategies. Molecular-level modifications of laccase can alter substrate accessibility, catalytic efficiency, structural stability, and surface properties, which subsequently influence enzyme–carrier interactions during immobilization. Conversely, the immobilization microenvironment can modulate the conformation, orientation, local stability, and operational performance of engineered laccases. Therefore, the rational combination of protein engineering and functional immobilization provides a synergistic approach for developing robust laccase biocatalysts with improved activity, stability, recyclability, and applicability under realistic wastewater treatment conditions.

Although laccase-catalyzed dye degradation, enzyme immobilization and nanomaterial-assisted wastewater treatment have been reviewed separately, an integrated strategy is still needed to connect dye molecular structure, enzyme engineering, immobilization design and wastewater-process validation. In contrast to previous reviews that have primarily summarized laccase sources, catalytic mechanisms, or individual immobilization technologies, this review adopts a cross-scale perspective that integrates molecular-level enzyme engineering with material-level immobilization strategies and process-level wastewater application. Specifically, the novelty of this review lies in establishing a structure–function–process framework that links dye molecular characteristics, laccase catalytic properties, engineered enzyme variants, immobilization microenvironments, and real wastewater treatment performance. In addition, particular emphasis is placed on the gap between laboratory-scale decolorization studies and practical implementation, including the limitations associated with complex wastewater matrices, mediator dependence, catalyst recovery, and long-term operational stability. This integrated perspective provides a more comprehensive roadmap for designing next-generation laccase-based systems for sustainable dye wastewater remediation. This review therefore focuses on engineered and immobilized laccase systems for dye wastewater treatment from a mechanism-to-engineering perspective. Recent advances during the past five years have substantially reshaped the development landscape of laccase-based dye wastewater treatment. In particular, progress in metagenomic mining has enabled the discovery of novel laccases with improved tolerance to harsh environmental conditions and broader dye degradation capabilities, expanding the available enzyme diversity for bioremediation applications (Ariaeenejad et al. 2023, 2024; Lima et al. 2022; Muhammad et al. 2024). Recombinant expression platforms have further facilitated the production, characterization, and application of laccases from diverse microbial sources, overcoming limitations associated with native enzyme availability (Huang et al. 2024; Sodhi et al. 2024; Huy et al. 2021; Jia et al. 2022). Meanwhile, protein engineering strategies, including rational design, directed evolution, and site-specific modification, have demonstrated significant potential for improving catalytic efficiency, substrate specificity, pH stability, and dye decolorization performance (Bian et al. 2024a, b; Li et al. 2025b). In parallel, artificial intelligence-assisted enzyme design and computational approaches are emerging as powerful tools for predicting enzyme structures, guiding functional optimization, and accelerating the development of high-performance laccase variants (Bibi et al. 2025; Barber-Zucker et al. 2022). Advanced immobilization materials, including metal–organic frameworks, graphene-based supports, biochar, hydrogels, and magnetic nanomaterials, have improved enzyme stability, recyclability, and operational feasibility in dye wastewater treatment systems (Alvarado-Ramírez et al. 2024; Chen et al. 2025). However, many recent studies remain focused primarily on laboratory-scale demonstrations, highlighting the need to critically evaluate whether these technological advances can be effectively translated into continuous, large-scale wastewater treatment processes (Dahiya et al. 2023; Brugnari et al. 2021).

Specifically, we compare how dye classes differ in their susceptibility to laccase oxidation, how fungal and bacterial laccases differ in environmental tolerance and substrate affinity, how rational design, directed evolution and semi-rational mutagenesis improve activity or stability, and how immobilization carriers affect laccase loading, retained activity, leakage, reusability and applicability in complex effluents. In addition, we distinguish single-dye model solutions, simulated wastewater, real textile wastewater and continuous reactor systems, and discuss why performance often decreases under real wastewater conditions. Furthermore, reported decolorization efficiencies across different studies cannot always be directly compared because experimental parameters, including initial dye concentration, enzyme dosage, reaction time, pH, temperature, mediator addition and analytical methods, vary substantially among investigations. Therefore, numerical removal percentages summarized in this review should be interpreted in their specific experimental contexts rather than as absolute indicators of superior catalytic performance. However, it should be recognized that most reported laccase-based dye degradation studies are conducted using individual synthetic dyes under controlled laboratory conditions, which provide valuable mechanistic insights but only partially represent the complexity of industrial textile wastewater. Real effluents contain mixtures of structurally diverse dyes, salts, surfactants, auxiliary chemicals, suspended solids and fluctuating physicochemical parameters, all of which may alter enzyme activity, substrate accessibility, mediator efficiency and immobilized-carrier performance. Therefore, laboratory-scale decolorization results should be interpreted primarily as indicators of catalytic potential rather than direct predictions of industrial treatment efficiency. However, it is noteworthy that the majority of reported laccase-mediated dye degradation studies remain at the laboratory scale, primarily using purified dyes or simulated wastewater systems under controlled conditions. Although these studies provide valuable mechanistic insights and demonstrate the catalytic potential of laccase-based systems, improved decolorization efficiency under model conditions does not necessarily translate into successful industrial implementation. Real textile wastewater contains complex mixtures of dyes, salts, surfactants, auxiliary chemicals, and potential enzyme inhibitors, which may substantially influence catalytic performance, enzyme stability, and treatment efficiency. Therefore, the transition from laboratory-scale validation to practical industrial application requires further assessment using real wastewater matrices, continuous operation systems, catalyst recovery strategies, and long-term performance evaluation. The transition from batch experiments to continuous treatment represents a major engineering challenge. Reactor configuration, hydraulic retention time, enzyme residence time, catalyst recovery, mass transfer efficiency and long-term operational stability can strongly influence laccase performance. Therefore, continuous-flow and pilot-scale studies provide essential evidence for determining whether laboratory-developed laccase systems can achieve stable and economically feasible wastewater treatment. To promote meaningful comparison among reported laccase systems, this review considers not only dye removal efficiency, but also critical experimental parameters, including enzyme source, dye concentration, reaction conditions, mediator requirement, immobilization strategy, operational stability and analytical verification of degradation products. Such standardized comparison is necessary because apparent differences in performance may arise from variations in experimental design rather than intrinsic differences in enzyme capability. By linking current limitations with future design strategies, this review provides an integrated framework for advancing laccase-based dye wastewater treatment.

## Degradation of dyes by laccase

### Basic principle of the laccase reaction

**Fig. 2:**
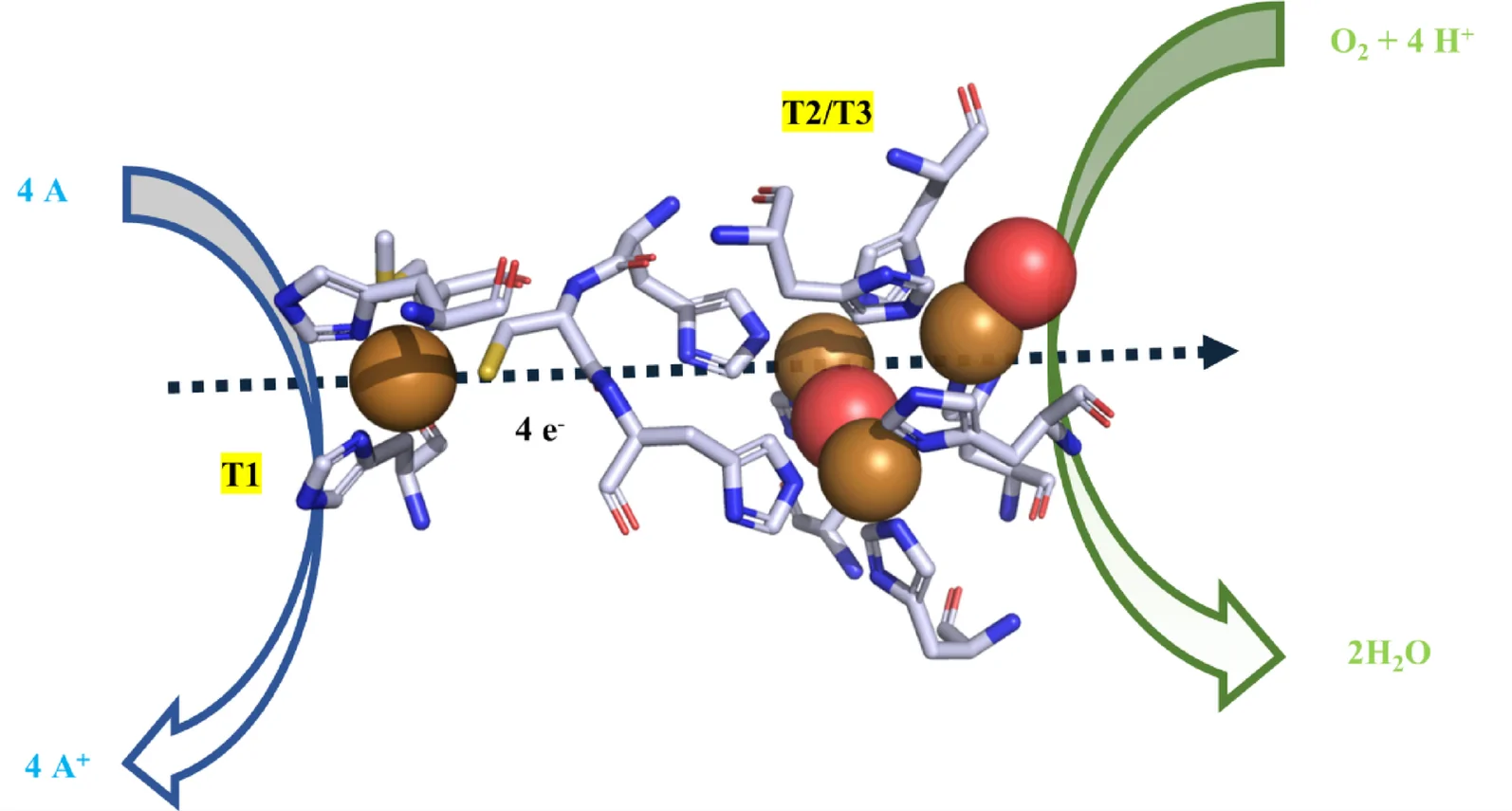
Mechanism of action in the catalytic centre of *Bacillus subtilis* laccase (PDB ID: 1GSK)

Laccases are multicopper oxidases whose catalytic function is based on electron transfer from organic substrates to molecular oxygen, resulting in the formation of water as the final reduction product (Fig. 2). For dye wastewater treatment, the important feature of the laccase catalytic cycle is not only the presence of copper centers, but also the ability of the enzyme to mediate oxidation of structurally diverse aromatic compounds through interactions between the substrate-binding region, redox potential and electron-transfer pathway.

These mechanistic characteristics provide the basis for understanding why different dye classes exhibit distinct oxidation behaviors and why enzyme engineering strategies targeting redox properties, substrate accessibility and catalytic stability can modify laccase performance for wastewater treatment applications.

### Laccase-catalyzed dye degradation mechanisms

It is noteworthy that the mechanistic pathways discussed in this section are derived from different levels of experimental evidence. Pathways directly supported by structural identification of transformation products (e.g., LC-MS/MS, GC-MS, NMR, or other molecular characterization techniques) are considered experimentally verified degradation routes. In contrast, mechanisms inferred primarily from changes in spectroscopic signals (such as UV–Vis or FTIR), color removal profiles, or degradation kinetics should be regarded as proposed pathways. This distinction is essential because decolorization indicates disruption of chromophoric structures, while does not necessarily confirm complete molecular degradation or identify specific reaction intermediates.

#### Decolorization mechanisms of laccases for azo dyes

Azo dyes, which are aromatic compounds featuring one or more azo bonds (-N = N-), are synthesized by the coupling of diazonium salts with coupling components such as amines or phenols. They are the most widely used class of synthetic dyes, accounting for approximately 70% of all commercial dyes (Dos Santos et al. 2007). The azo bond is typically flanked by benzene or naphthalene rings, which may bear substituents such as -Cl, -CH_3_, -NO_2_, -NH_2,_ -OH, and -CO. These groups influence key properties, including polarity, solubility, thermal stability, and lightfastness, with donor substituents (e.g. -NH_2_ and -OH) generally enhancing biodegradability under reducing conditions and electron-withdrawing groups (e.g. -NO_2_) providing increased stability and resistance to degradation (Carrascal-Hernández et al. 2025). Common dyes include CR, MO, xylidine, and Reactive Blue 52 (Brown and De Vito 1993; Banat et al. 1996).

Laccase directly catalyzes single-electron transfer in azo dye molecules, particularly in those with electron-donating groups such as phenolic hydroxyl or amino groups, generating unstable radical intermediates that initiate chain reactions leading to azo bond cleavage. Although azo-bond cleavage is strongly supported by the disappearance of characteristic azo-related spectral signals and identification of aromatic transformation products in some LC-MS analyses, the detailed radical-mediated sequence remains system-dependent and should be considered a proposed mechanistic interpretation unless transient intermediates are directly detected. Therefore, the reported pathway represents a generally accepted oxidative model supported by multiple analytical observations rather than a universally confirmed reaction sequence for all azo dyes. These intermediates can undergo further oxidation to form polyphenolic compounds with benzene ring structures, which are then degraded through additional dehydrogenation of the aromatic compounds into smaller, colorless or low-toxicity products, thereby achieving decolorization (Selvaraj et al. 2021; Abd El-latif et al. 2024). This degradation mechanism is well illustrated by the transformation of the monoazo dye Acid Red 27 (AR 27) by *Bacillus drentensis* laccase. The laccase attacks and cleaves the azo bond (-N = N-), destroying the chromophore structure. The oxidation reactions further promote ring opening within the dye molecule, resulting in a significant decrease in color intensity. Under incubation at room temperature for 5 h, the laccase degraded 50 ppm AR 27 and Direct Blue 6 with 99.7% and 67.43% efficiency, respectively (Jeyabalan et al. 2025). Liquid chromatography–mass spectrometry (LC-MS) analysis identified transformation products such as benzoic acid and aminosulfonic naphthalene derivatives, supporting azo-bond cleavage and subsequent fragmentation of the dye structure. Similarly, laccase from *Oudemansiella canarii* decolorized 80% of 50 mg/L CR within 24 h at 30 °C and pH 5.5 (Iark et al. 2019). The disappearance of azo- and aromatic-ring-related Fourier-transform infrared spectrometry (FTIR) bands, together with the appearance of carboxyl-associated signals, indicated azo-bond disruption and formation of oxidized products. Nevertheless, FTIR-based evidence should be interpreted together with molecular-level analyses because changes in functional-group signals alone cannot determine the identity or toxicity of transformation products. LC-MS or GC-MS analyses are therefore particularly valuable for confirming cleavage products and tracking sequential oxidation pathways, while TOC measurements and toxicity assays provide complementary evidence regarding mineralization and environmental safety.

**Fig. 3:**
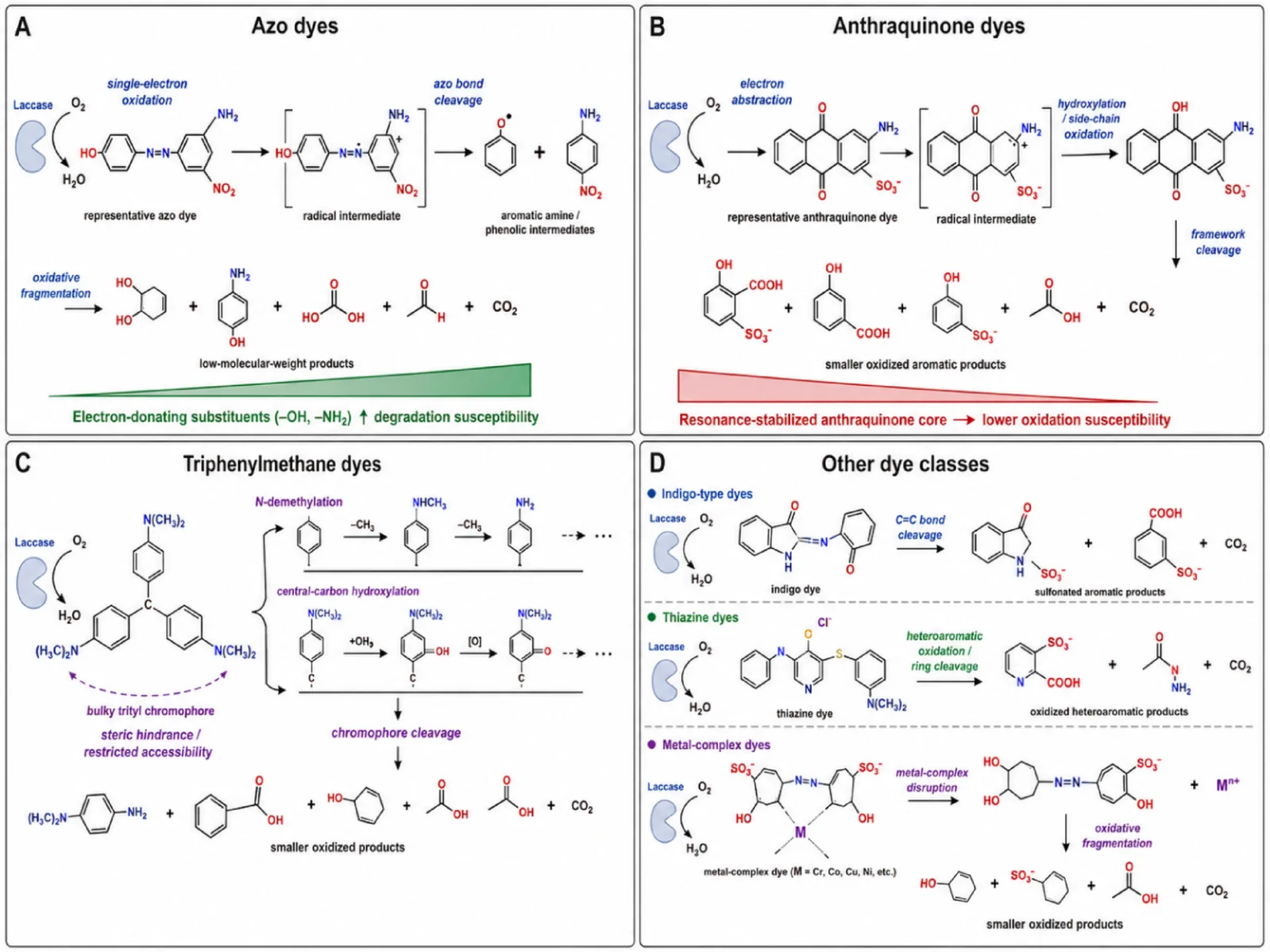
Representative laccase-mediated degradation pathways of major dye classes. **A** Azo dyes. **B** Anthraquinone dyes. **C** Triphenylmethane dyes. **D** Other dye classes, including indigo, thiazine, and metal-complex dyes. The schematics summarize the principal oxidative transformation routes catalyzed by laccase, including radical formation, chromophore cleavage, hydroxylation, N-demethylation, and subsequent fragmentation into smaller products

The representative oxidation pathway of azo dyes is summarized in Fig. 3A. This schematic illustrates the major steps involved in laccase-mediated azo dye degradation, including single-electron oxidation, azo bond cleavage, formation of aromatic intermediates and subsequent oxidative fragmentation. The proposed degradation routes of anthraquinone dyes are illustrated in Fig. 3B. The schematic emphasizes the initial electron abstraction step, radical intermediate formation, hydroxylation reactions and cleavage of the anthraquinone framework, which together explain the lower oxidation susceptibility of resonance-stabilized anthraquinone structures compared with azo dyes. The main oxidation pathways of triphenylmethane dyes are summarized in Fig. 3C. The proposed mechanism highlights the influence of the bulky trityl chromophore on substrate accessibility and shows how N-demethylation, central-carbon hydroxylation and chromophore cleavage contribute to disruption of the conjugated color structure. Representative degradation pathways of other dye classes, including indigo, thiazine and metal-complex dyes, are summarized in Fig. 3D. Unlike azo, anthraquinone and triphenylmethane dyes, these structures undergo more diverse transformations, including C = C bond cleavage, metal-complex disruption and heteroaromatic ring oxidation.

#### Decolorization mechanisms of laccases for anthraquinone dyes

The chemical properties of anthraquinone dyes stem from their structural characteristics: an anthracene molecule composed of three fused benzene rings, with two carbonyl groups attached to the central ring (Yadav et al. 2021). These properties, combined with the resonance effect within the anthraquinone molecule, confer high chemical stability to anthraquinone dyes, making them difficult to degrade (Varjani et al. 2020). Representative members of this dye family include Remazol Brilliant Blue R (RBBR), Alizarin Green (AG), and Acid Blue 45 (Soares et al. 2001; Vikrant et al. 2018; Zhai et al. 2024).

The degradation of anthraquinone dyes by laccase is primarily initiated by an electron abstraction process that generates unstable radical intermediates, followed by oxidation, hydroxylation, and deamination reactions, which ultimately lead to the cleavage of the aromatic ring structure (Routoula and Patwardhan 2020; Dauda and Erkurt 2020). RBBR is a representative anthraquinone dye for elucidating this pathway. Pype et al. (2019) showed that laccase cleaves the 22-carbon RBBR parent molecule into 14-carbon and 8-carbon intermediates, indicating a defined carbon-chain scission step during anthraquinone degradation. This pathway suggests that laccase first destabilises the conjugated anthraquinone structure through oxidative attack, followed by cleavage of the chromophoric framework and formation of smaller aromatic intermediates. For anthraquinone dyes, LC-MS and HPLC analyses provide direct evidence for the formation of lower-molecular-weight transformation products and support oxidative fragmentation of the parent structure. However, specific reaction steps, including the order of hydroxylation, side-chain oxidation, and aromatic-ring cleavage, are often reconstructed from detected intermediates and chemical plausibility rather than directly observed. These pathways should therefore be interpreted as experimentally supported models rather than complete mechanistic sequences. Consistently, Bibi et al. (2025) reported that laccase from *Geobacillus stearothermophilus* MB600 transformed RBBR within 4 h at 75 °C, with FTIR and High Performance Liquid Chromatography (HPLC) analyses showing new functional-group signals and multiple product peaks, further supporting oxidative fragmentation of the parent dye. For AG, Peng et al. (2021) propose a C-α oxidation pathway. Electron abstraction through the T1 Cu was suggested to initiate cleavage between the anthraquinone core and side chain, while residues including Glu460, Phe85 and Leu58 contributed to substrate positioning. The system removed 95.86% of AG within 12 h, illustrating how active-site interactions can direct anthraquinone dye oxidation.

#### Decolorization mechanisms of laccases for triphenylmethane dyes

Triphenylmethane dyes contain a central carbon atom linked to three aromatic rings and include MG, CV, Acid Violet 17 (AV 17), Brilliant Green (BG) and Methyl Violet (Varjani et al. 2020; Li et al. 2025a). Their bulky trityl chromophore contributes to high structural stability and can restrict access to the laccase active site. Therefore, triphenylmethane dyes are often more difficult to oxidize directly than structurally simpler dyes and may show stronger mediator dependence.

Laccase degrades triphenylmethane dyes mainly via two pathways: N-demethylation and conjugated chromophore structural cleavage. The proposed N-demethylation and central-carbon oxidation pathways are supported by the identification of characteristic intermediates, including Michler’s ketone derivatives and demethylated products in LC-MS analyses. Nevertheless, the relative contribution of these pathways may vary among different triphenylmethane dyes and laccase systems. In many cases, the proposed sequence of intermediate formation remains a mechanistic interpretation based on product profiles rather than direct observation of individual reaction steps. The first pathway starts with sequential N-demethylation, which disrupts the stability of the conjugated system, resulting in chromophore cleavage (Rashtbari and Dehghan 2021). The second pathway commences with the hydroxylation of the dye molecule, yielding an unstable methanol intermediate that rapidly decomposes, thereby destroying the conjugated structure. These pathways may operate concurrently or preferentially, depending on the laccase source, dye structure, and reaction conditions. MG degradation by Trametes cubensis laccase provides a representative example of the central-carbon hydroxylation pathway. Himanshu et al. (2025) proposed that hydroxylation at the central carbon generates Michler’s ketone, followed by deamination and oxidative cleavage of aromatic substituents, leading to progressive disruption of the triphenylmethane framework. A comparable pathway has also been reported for CV. A recombinant laccase CotA from *Bacillus amyloliquefaciens* efficiently degraded CV into bis[4-(dimethylamino) phenyl] methanone and its N-demethylation derivative via central carbon hydroxylation and subsequent cleavage of the conjugated chromophore (Yang et al. 2020a).

#### Decolorization mechanisms of laccases for other types of dyes

In addition to azo, anthraquinone and triphenylmethane dyes, laccases can also transform indigo, thiazine and metal-complex dyes. Because these dyes contain distinct chromophoric structures, their degradation routes are more diverse and cannot be described by a single universal pathway. Ruxandra Leontieș et al. (2022) investigated laccase-mediated degradation of Naphthol Green B (NGB) and Indigo Carmine (IC). High-resolution mass spectrometry suggested that NGB underwent oxidative cleavage accompanied by iron-complex disruption and naphthalene-ring transformation. By contrast, IC degradation mainly involved cleavage of the central C = C bond and formation of sulfonated aromatic products. These findings indicate that metal-complex and indigo dyes can follow different degradation routes even within the same laccase-based system. Hordieieva et al. (2023) further clarified the degradation route of IC using a *Trametes versicolor* (*T. versicolor*) laccase/TEMPO/O₂ system. HPLC monitoring revealed the complete disappearance of the IC peak and the formation of key intermediates including isatin-5-sulfonic acid, 4-sulfo-3-anthranilic acid, and anthranilic acid, suggesting a three-stage mechanism involving oxidative cleavage, decarboxylation, and desulfonation. Methylene Blue (MB), a representative thiazine dye, contains an N–S-conjugated heteroaromatic chromophore. Salem et al. (2024) reported a laccase from *T. harzianum* AUMC14893 that degrades MB through a sequential oxidative mechanism, involving an initial attack on the central aromatic ring, followed by breakage of the side-chain aromatic rings, generating small-molecular products such as R–NH_3_^+^, resulting in decolorization.

For the three major dye classes—azo, anthraquinone and triphenylmethane dyes—as well as other dye types, laccase-catalyzed degradation is governed by chromophore architecture, substituent effects, molecular size and redox properties. Azo dyes are generally more susceptible to laccase oxidation, particularly those bearing electron-donating groups such as –OH or –NH₂, which facilitate electron transfer to the T1 Cu site. Single-electron oxidation, radical formation and azo-bond cleavage are the dominant routes, with aromatic amine or phenolic intermediates persisting when subsequent oxidation is incomplete. Anthraquinone dyes are less readily fragmented because the fused quinone–aromatic scaffold is resonance-stabilised; degradation usually involves electron abstraction, hydroxylation and side-chain cleavage, followed by slower partial ring opening. Triphenylmethane dyes are often the most recalcitrant among these classes, as bulky triaryl frameworks and highly conjugated central-carbon chromophores restrict active-site access and stabilise oxidation intermediates. Stepwise N-demethylation or N-deethylation, central-carbon hydroxylation and disruption of the trityl conjugated system are therefore the main transformation routes. Other dye types follow structure-dependent oxidative pathways, with sterically hindered, high-redox-potential or poorly accessible substrates showing greater dependence on mediator-assisted oxidation. Overall, laccase-catalyzed dye degradation is a chromophore-dependent oxidative process in which dye structure determines the initial attack site, intermediate formation and subsequent fragmentation pathway. To provide an integrated comparison of laccase-mediated degradation mechanisms among different dye classes, Table 1 summarizes the structural characteristics, dominant oxidation pathways, influencing factors, analytical evidence and major limitations associated with each dye category. This comparison highlights that laccase performance is determined not only by enzyme properties but also by the chemical architecture and accessibility of the target dye molecules. Although individual studies demonstrate high decolorization efficiencies for specific dyes, direct comparison among reported percentages requires caution. For example, apparent differences in degradation efficiency may originate from variations in dye concentration, enzyme activity units, substrate accessibility, reaction duration or the presence of mediators rather than intrinsic differences in laccase catalytic capability. Therefore, mechanistic comparisons should concentrate primarily on substrate susceptibility, oxidation pathways and enzyme–dye interactions, while numerical efficiency values should be considered under the corresponding experimental conditions.

**Table 1:** Comparison of laccase-mediated degradation mechanisms for different dye classes

Dye class	Representative dyes	Structural characteristics	Main laccase oxidation pathway	Key influencing factors	Common analytical evidence	Main limitation
Azo dyes	Congo Red, Methyl Orange, Acid Red G	–N=N– azo bond connecting aromatic rings	Single-electron oxidation → radical formation → azo bond cleavage → aromatic fragmentation	Electron-donating groups enhance oxidation; electron-withdrawing groups increase resistance	LC-MS, FTIR, HPLC, toxicity assays	Aromatic amine intermediates may persist
Anthraquinone dyes	RBBR, Alizarin Green, Acid Blue	Fused anthraquinone ring with carbonyl groups	Electron abstraction → hydroxylation → side-chain cleavage → partial ring opening	Resonance stabilization, redox potential, steric accessibility	LC-MS, HPLC, FTIR, NMR	High structural stability
Triphenylmethane dyes	Malachite Green, Crystal Violet	Three aromatic rings linked by central carbon	N-demethylation → central carbon hydroxylation → chromophore cleavage	Bulky structure, active-site accessibility, mediator dependence	LC-MS, HPLC, FTIR	Strong steric hindrance
Other dyes	Indigo, thiazine, metal-complex dyes	Diverse heteroaromatic or metal-containing structures	C=C cleavage, heterocycle oxidation, metal-complex disruption	Metal coordination, molecular size, charge	HR-MS, LC-MS, FTIR, toxicity assays	Highly structure-dependent pathways

Although single-dye experiments have remarkably improved the understanding of structure-dependent laccase oxidation pathways, their interpretation should be extended cautiously to industrial wastewater treatment. In textile effluents, multiple dyes with different redox potentials, molecular sizes and functional groups coexist and may compete for the laccase active site or mediator-derived radicals. In addition, non-dye components, such as salts, surfactants and residual processing chemicals may inhibit catalytic activity or modify dye–enzyme interactions. Consequently, degradation pathways identified using purified dye solutions represent fundamental reaction models, whereas mixed-dye and real-effluent studies are required to evaluate competitive effects, transformation efficiency and treatment robustness under practical conditions.

Beyond chromophore classification, the molecular architecture of dye substrates critically determines their susceptibility to laccase oxidation through multiple interconnected factors, including steric accessibility, electron density distribution, and redox potential compatibility. Substrates with accessible electron-rich aromatic regions are generally more favorable for oxidation because they facilitate electron transfer from the dye molecule to the laccase active site. In contrast, bulky substituents, sterically shielded chromophores, and highly condensed aromatic structures may reduce substrate accessibility and hinder productive enzyme–substrate interactions, thereby decreasing catalytic efficiency.

The redox potential difference between the laccase catalytic center and the dye substrate is another important determinant of oxidation efficiency. High-redox-potential laccases generally exhibit broader oxidative capacity toward structurally stable dyes, whereas low-redox-potential laccases may show limited activity toward substrates with high oxidation barriers. However, catalytic performance is not governed solely by thermodynamic factors, as substrate binding orientation, hydrophobic interactions, and active-site accessibility also strongly influence reaction rates. Therefore, efficient degradation of structurally diverse dyes requires coordinated optimization of both enzyme properties and substrate-specific catalytic interactions.

Although different dye classes undergo laccase-mediated degradation through partially overlapping oxidative mechanisms, their molecular structures strongly influence reaction efficiency, degradation pathways, and the requirements for enzyme optimization. Azo dyes, characterized by one or more azo bonds (–N = N–), are primarily degraded through oxidative cleavage of azo linkages, resulting in the disruption of chromophoric structures and subsequent formation of aromatic intermediates. However, the presence of electron-withdrawing or sterically hindered substituents may reduce accessibility and limit degradation efficiency. Anthraquinone dyes generally exhibit higher structural stability due to their fused aromatic quinone frameworks, requiring stronger oxidative conditions or mediator-assisted systems to achieve efficient transformation. Triphenylmethane dyes, containing extended conjugated aromatic structures, are often susceptible to laccase-mediated oxidation through radical formation and chromophore disruption, although steric effects and intermediate toxicity may influence complete mineralization. Other dye classes, including indigoid, phthalocyanine, and heterocyclic dyes, display diverse degradation behaviors depending on their electronic structures, metal coordination, and substituent patterns.

From a comparative perspective, azo dyes are generally more responsive to direct oxidative cleavage of chromophoric bonds, whereas anthraquinone and highly conjugated dyes often require enhanced catalytic systems, including redox mediators, engineered laccases with improved substrate affinity, or immobilization strategies that promote favorable enzyme–substrate interactions. Therefore, dye molecular architecture should be considered a critical parameter when designing engineered laccase systems, selecting immobilization carriers, and predicting treatment performance under realistic wastewater conditions. Verification of these proposed degradation pathways requires integration of multiple analytical techniques. UV–visible spectroscopy is commonly used for monitoring chromophore disappearance and decolorization, while it cannot confirm molecular breakdown or mineralization. LC-MS and GC-MS provide detailed information on transformation products and allow identification of intermediate compounds generated during oxidation, whereas FTIR analysis reveals changes in functional groups associated with bond cleavage, oxidation or formation of new chemical structures. However, detection of degradation intermediates alone does not demonstrate complete pollutant removal; therefore, mineralization indicators such as total organic carbon (TOC) reduction and toxicity assays are necessary to evaluate whether oxidation products are less persistent and less harmful than the original dyes.

### Mediator systems

Mediator systems partially overcome substrate limitations by acting as diffusible redox intermediates that expand the oxidative range of laccases. This strategy is particularly relevant for dyes with high redox potentials or sterically inaccessible chromophores that cannot be efficiently oxidized through direct enzyme–substrate electron transfer. However, mediator selection should consider not only oxidation efficiency, but also mediator stability, potential toxicity, cost, and suitability for large-scale wastewater treatment.

Synthetic and natural mediators differ substantially in catalytic efficiency, environmental compatibility, and practical applicability. Synthetic mediators, including 2,2′-azino-bis(3-ethylbenzothiazoline-6-sulfonic acid) (ABTS), 1-hydroxybenzotriazole (HBT), and 2,2,6,6-tetramethylpiperidine-1-oxyl (TEMPO), generally exhibit high redox activity and broad substrate oxidation capability, enabling efficient degradation of structurally recalcitrant dyes with high oxidation potentials. However, their large-scale application is restricted by relatively high cost, limited sustainability, potential toxicity of oxidation products, and concerns regarding environmental release. In contrast, natural mediators derived from lignin degradation products or plant secondary metabolites, such as syringaldehyde, acetosyringone, and related phenolic compounds, provide advantages in terms of biodegradability, lower environmental impact, and improved compatibility with sustainable wastewater treatment processes. Nevertheless, natural mediators often exhibit lower catalytic efficiency, weaker radical generation capacity, and greater variability depending on substrate structure and reaction conditions. Therefore, the selection of mediators requires balancing oxidative performance with environmental and economic considerations. Future industrial applications may benefit from the development of low-toxicity, renewable mediator systems or hybrid strategies combining engineered laccases with optimized natural mediators.

**Fig. 4:**
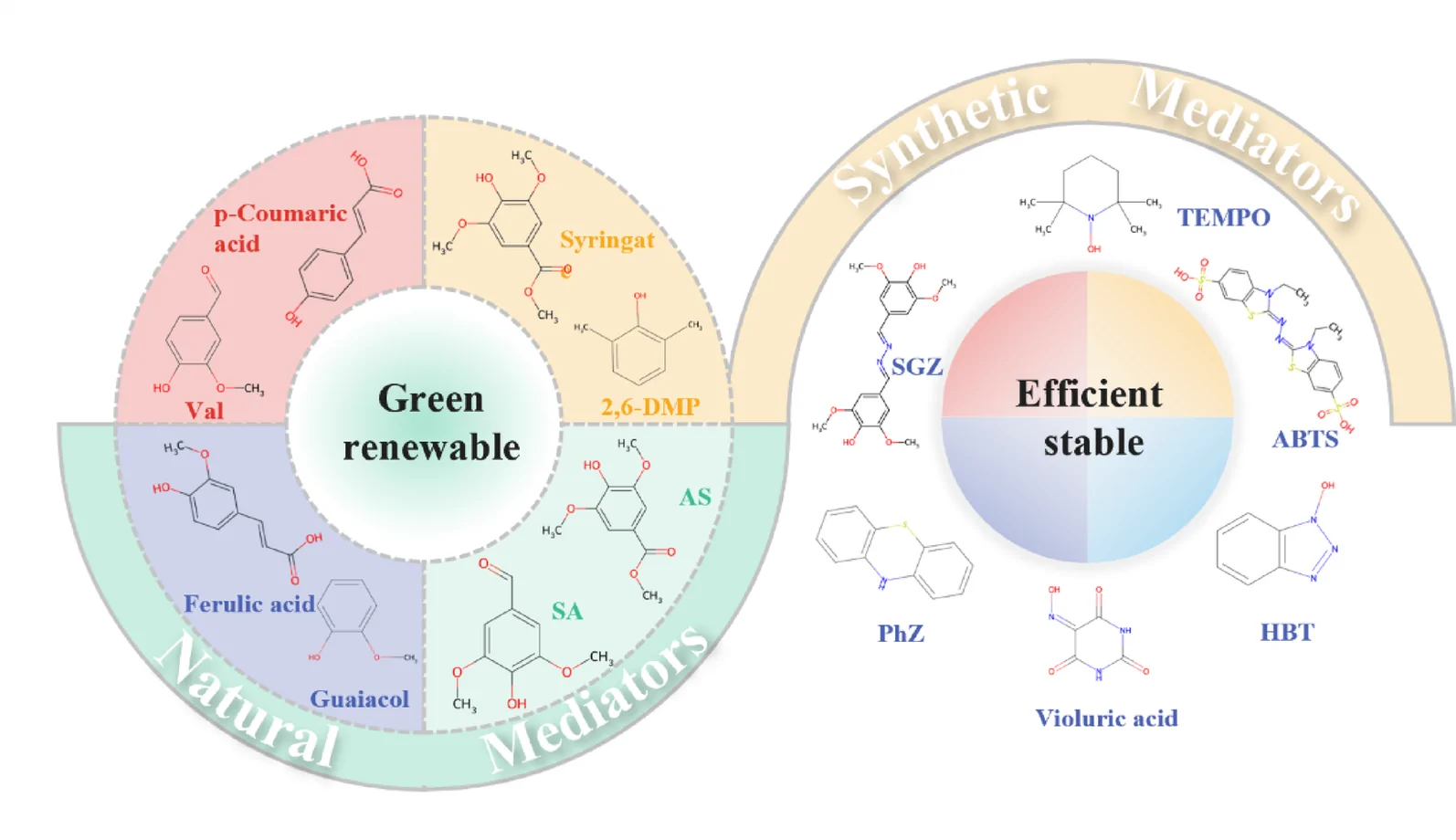
Structures of natural and synthetic mediators

Although laccases can directly oxidize many phenolic and electron-rich dyes, their activity towards bulky, non-phenolic or high-redox-potential dyes is often limited by steric hindrance and inefficient electron transfer (Coria-Oriundo et al. 2021). These constraints can be overcome by employing redox mediators, which, upon oxidation by laccase, generate diffusible radical intermediates that act as electron shuttles to attack dye chromophores, thereby significantly enhancing the degradation efficiency. Laccase mediators are commonly categorised into natural and synthetic mediators (Fig. 4). Natural mediators include p-coumaric acid, vanillin (Val), ferulic acid, guaiacol, syringaldehyde (SA), acetosyringone (AS), syringate, and 2,6-dimethylphenol (2,6-DMP). Synthetic mediators include ABTS, HBT, TEMPO, violuric acid, phenothiazine (PhZ), and syringaldazine (SGZ) (Asadi et al. 2020; Li et al. 2022a).

The effect of mediators is evident when synthetic and natural mediator systems are compared. For *T. versicolor* laccase, Orange G (OG) and Ponceau 4R (P4R) showed only limited decolorization in the absence of mediators, whereas ABTS increased decolorization to more than 40% within 15 min at pH 4.0 and 25 °C; the corresponding *k*_cat_ values increased by approximately 200-fold for OG and 300-fold for P4R (Vacalie et al. 2024). This case highlights the capacity of ABTS to accelerate electron transfer between laccase and structurally resistant azo dyes. Natural phenolic mediators can also be effective: AS and SA enabled more than 90% decolorization of multiple dyes within 4 h at pH 4.0 and 60 °C when used at 0.1 mM (Sun et al. 2021). These examples show that laccase–mediator systems can extend the oxidative range of laccases beyond active-site accessibility and intrinsic redox potential, particularly for bulky, non-phenolic or high-redox-potential dyes that are poorly oxidized by laccase alone. Yet enhanced decolorization in model dye solutions should not be directly equated with process feasibility, as mediator cost, toxicity, secondary oxidation products, radical scavenging by wastewater constituents, and mediator recovery or retention remain important constraints. Future laccase–mediator systems should therefore be evaluated not only by decolorization efficiency, but also by mediator dose, reusability, product safety and performance in complex wastewater matrices. Compared with chemical oxidation technologies, such as ozonation, Fenton oxidation and photocatalysis, laccase–mediator systems provide a biologically driven alternative that can operate under milder conditions and avoid excessive chemical consumption. However, physicochemical oxidation processes generally achieve faster degradation and broader pollutant oxidation, whereas laccase systems provide greater selectivity that may require longer reaction times and optimized enzyme–mediator combinations. Therefore, laccase-based oxidation should be considered complementary to, rather than universally replacing, conventional advanced oxidation processes.

Despite their ability to enhance laccase-mediated oxidation, mediator-assisted systems face several challenges that limit their large-scale implementation. First, mediator recovery and recycling remain technically challenging, particularly for low-molecular-weight mediators that are difficult to separate from treated wastewater, leading to increased material consumption and process complexity. Second, incomplete oxidation or transformation of mediators may generate secondary products with unknown toxicity profiles, raising concerns regarding potential environmental impacts after wastewater discharge. This issue is particularly relevant for synthetic mediators, whose aromatic structures and oxidation-derived intermediates require comprehensive toxicity evaluation.

From an operational perspective, mediator-assisted processes introduce additional costs associated with mediator production, supplementation, recovery, and downstream treatment. The requirement for continuous mediator input may significantly affect the economic feasibility of large-scale applications compared with mediator-free or immobilized enzyme systems. Furthermore, regulatory considerations related to chemical additives, residual mediator concentrations, and potential ecological effects must be addressed before industrial deployment. Therefore, future development should focus on renewable, low-toxicity mediator alternatives, efficient mediator recovery strategies, and integrated reactor designs that minimize mediator consumption while maintaining high catalytic performance.

## Development and modification of laccase molecules

### New and recombinant laccases

Novel laccases with unique catalytic properties are continually identified from diverse sources, facilitating adaptation to various reaction environments (Sharma et al. 2018). Further, heterologous expression of fungal and bacterial laccases using recombinant technologies has emerged as a highly effective strategy to enhance enzymatic performance and yield. In the context of dye wastewater treatment, recombinant expression is not only a strategy to increase enzyme availability, but also a prerequisite for generating sufficient quantities of laccases with consistent catalytic properties for decolorization applications (Bannach-Machado et al. 2026). By enabling controlled production of selected laccase variants, heterologous expression facilitates systematic evaluation of dye oxidation capacity, substrate specificity and operational stability under wastewater-relevant conditions.

#### Fungal laccases

Fungal laccases represent the most extensively explored group of dye-decolorizing oxidases. Their generally high redox potential and broad substrate range make them effective against structurally diverse aromatic dyes, but their sensitivity to temperature, pH and production conditions limits direct process application (Aza and Camarero 2023). Representative laccase-producing fungal species encompass *T. versicolor*, *Trametes pubescens*, *Pleurotus ostreatus*, *Phlebia brevispora*, *Coprinopsis cinerea*, and *Laetiporus bicolor* (Strong and Claus 2011; El-Gendi et al. 2021). Recent fungal laccase mining has therefore focused less on discovering another active enzyme and more on expanding the operational window of this enzyme class (Shoda et al. 2016; Wiśniewska et al. 2021a).

Lac37 II from *Trametes trogii* S0301 exemplifies the value of fungal laccase mining for improving thermal robustness. At 60 °C, Lac37 II decolorized 25 mg/L MG and CV by 100% and 99.1%, respectively, within 5 h (Yang et al. 2020b), while retaining approximately 50% residual activity after 12 h at the same temperature. Its enhanced thermostability was associated with two loop-region proline residues, P13 and P351, suggesting that local structural rigidity may contribute to sustained catalytic performance under elevated-temperature conditions. This exceptional thermostability provides a solid basis for sustained decolorization under high-temperature conditions. Other fungal laccases define complementary but more specific advantages. The cold-adapted G3 IBMiP from *Kabatiella bupleuri* retained 90% activity after 2 h at 10 °C, although its decolorization of 250 mg/L CV and Basic Fuchsin (BF) reached only 40.4% and 31.7% within 1 h (Wiśniewska et al. 2021b). Its value therefore lies in low-temperature stability rather than high dye-removal efficiency. *T. zonata* showed the highest extracellular laccase production, and Cu^2+^ supplementation further increased enzyme activity by up to 38-fold. The purified laccase showed broad substrate specificity and achieved up to 95% mediator-free decolorization of synthetic azo dyes, indicating that strain screening can identify fungal laccases with both production advantages and reduced mediator dependence (Demkiv et al. 2026). Crude laccase from *Pleurotus ostreatus* 50,711 decolorized and detoxified single and mixed azo, anthraquinone, triphenylmethane and indigo dyes, retaining activity during continuous-batch treatment (Lai et al. 2026).

#### Bacterial laccases

Bacterial laccases have been identified from diverse genera, including *Bacillus pumilus*, *B. subtilis*, *Bacillus licheniformis*, *Streptomyces lavendulae*, *Streptomyces griseus*, *Escherichia coli*, *Pseudomonas syringae*, *Thermus thermophilus*, and *Sinorhizobium meliloti* (Minussi et al. 2002; Shoda et al. 2016). Compared with many fungal laccases, they generally show stronger tolerance to high temperature, alkaline pH and salinity, although their oxidative capacity toward high-redox-potential substrates is often more limited (Guan et al. 2018; Mardiyono et al. 2026). This trade-off makes bacterial laccases particularly relevant when dye decolorization is constrained by harsh reaction conditions rather than by substrate oxidation alone. Recent studies have therefore focused on mining bacterial laccases from environmentally stressed habitats, such as tannery, textile wastewater or thermophilic environments, to obtain enzymes with improved stability, broader pH adaptability or reduced mediator dependence (Janusz et al. 2020).

Metagenomic approaches have emerged as powerful strategies for expanding the discovery of novel laccases beyond the limitations of conventional cultivation-based screening. Since a large proportion of environmental microorganisms remain uncultivable under standard laboratory conditions, traditional isolation approaches may overlook diverse laccase sequences with unique catalytic properties. Functional and sequence-based metagenomic analyses enable direct exploration of microbial communities from extreme environments, including saline habitats, hot springs, contaminated soils and industrial wastewater ecosystems, where microorganisms may possess laccases adapted to high temperature, salinity, pH fluctuations or toxic compounds. Compared with conventional enzyme discovery, metagenomics provides access to a broader genetic reservoir and allows identification of previously unknown laccase families, including enzymes with unusual sequence characteristics or environmental adaptations. For example, PersiLac3 from a tannery metagenome is a strong example of this stress-adapted strategy. It decolorized CR by more than 95% at 1000–4000 mg/L and retained 82% decolorization even at 5000 mg/L within 15 min (Ariaeenejad et al. 2024). Salt further increased decolorization by approximately 10%, and treated CR showed reduced toxicity. This combination of high dye loading, salt responsiveness and toxicity-related evidence makes PersiLac3 more convincing than bacterial laccases tested only against low-concentration model dyes under mild conditions. Nevertheless, metagenomic discovery also presents several challenges. Sequence-based annotation remains dependent on the accuracy of available laccase databases and may incorrectly assign copper oxidases or related multicopper proteins as functional laccases. In addition, numerous metagenome-derived genes require heterologous expression optimization because their native hosts, secretion systems, folding machinery or post-translational modification pathways are unknown. Functional screening of metagenomic libraries is therefore essential to confirm catalytic activity, substrate specificity and environmental tolerance.

LfLAC3 from *Lysinibacillus fusiformis* provides a complementary example of thermal and pH robustness. It showed an optimum temperature of 70 °C, retained more than 70% activity at 100 °C, and decolorized seven dyes by 39%–70% at pH 7.0 (Zhang et al. 2023). Its main value therefore lies in environmental stability rather than exceptionally high decolorization efficiency. *Bacillus aestuarii* KSK, isolated from textile dye wastewater sediments, achieved 80% mediator-free MG decolorization within 20 min, whereas the native laccase from thermophilic *Bacillus licheniformis* SO8 decolorized Acid Red 27 by 38% and showed optimal activity at pH 9.0 and 70 °C (Selvam et al. 2022; Yıldırgan et al. 2026). New laccase mining is moving from the simple identification of dye-active enzymes towards the selection of enzymes with defined operational niches.

Fungal and bacterial laccases exhibit complementary biochemical characteristics that influence their suitability for different dye-treatment scenarios. Fungal laccases generally possess higher T1 Cu redox potentials and broader oxidation capability toward aromatic and high-redox-potential dye molecules, which contributes to their superior performance in degrading structurally complex substrates. However, their industrial application is frequently limited by lower tolerance to alkaline conditions, elevated temperatures, and high ionic strength environments commonly encountered in textile wastewater. In contrast, bacterial laccases typically display greater environmental robustness, including improved resistance to thermal stress, alkaline pH, and salinity, making them attractive candidates for harsh wastewater conditions. Nevertheless, their comparatively lower redox potential and narrower substrate oxidation range may restrict direct oxidation of some recalcitrant dyes.

From an industrial perspective, neither fungal nor bacterial laccases represent universally superior catalysts; instead, their advantages correspond to different application requirements. Fungal laccases are particularly suitable for high-efficiency oxidation of diverse aromatic pollutants under controlled conditions, whereas bacterial laccases may offer advantages in continuous treatment systems requiring operational stability under fluctuating environmental conditions. Future laccase development should therefore focus not only on identifying highly active enzymes but also on matching biochemical properties, including redox potential, substrate affinity, thermal stability, pH tolerance, and mediator dependence, with the specific characteristics of industrial wastewater streams.

#### Recombinant laccases

Recombinant expression addresses the supply and recovery limitations of native laccases (Sodhi et al. 2024). Many natural laccases show useful catalytic properties but are limited by low yield, unstable secretion or difficult purification (Yang et al. 2017). Heterologous expression can improve soluble production, extracellular release and compatibility with further protein engineering (Bannach-Machado et al. 2026). The mutant CotA/SF expressed in *Escherichia coli* provides a useful example of recombinant expression improving enzyme accessibility without losing dye-oxidizing activity. Co-expression with phospholipase C promoted extracellular release of the enzyme, while CotA/SF retained more than 80% activity after 10 h across pH 6.0–10.0, and achieved more than 70% MG decolorization under neutral-to-alkaline conditions (Xu et al. 2020). Recombinant CotA laccase from *Bacillus clausii* was produced in *E. coli* as catalytically active inclusion bodies. The 40EL-tagged biocatalyst showed enhanced activity and efficiently decolorized Eriochrome Black T and CR, particularly in the presence of redox mediators (Romero-Martínez et al. 2026). TP-Lac2 from *Saccharomyces cerevisiae* was secreted through its native signal peptide and achieved 94.6% decolorization of both Amido Black 10B and MG within 5 h at 50 °C when AS was supplied (Bucchieri et al. 2024). Recombinant expression links laccase production with functional optimisation, allowing laccases to be tailored for improved secretion, stability and dye-decolorization performance.

**Fig. 5:**
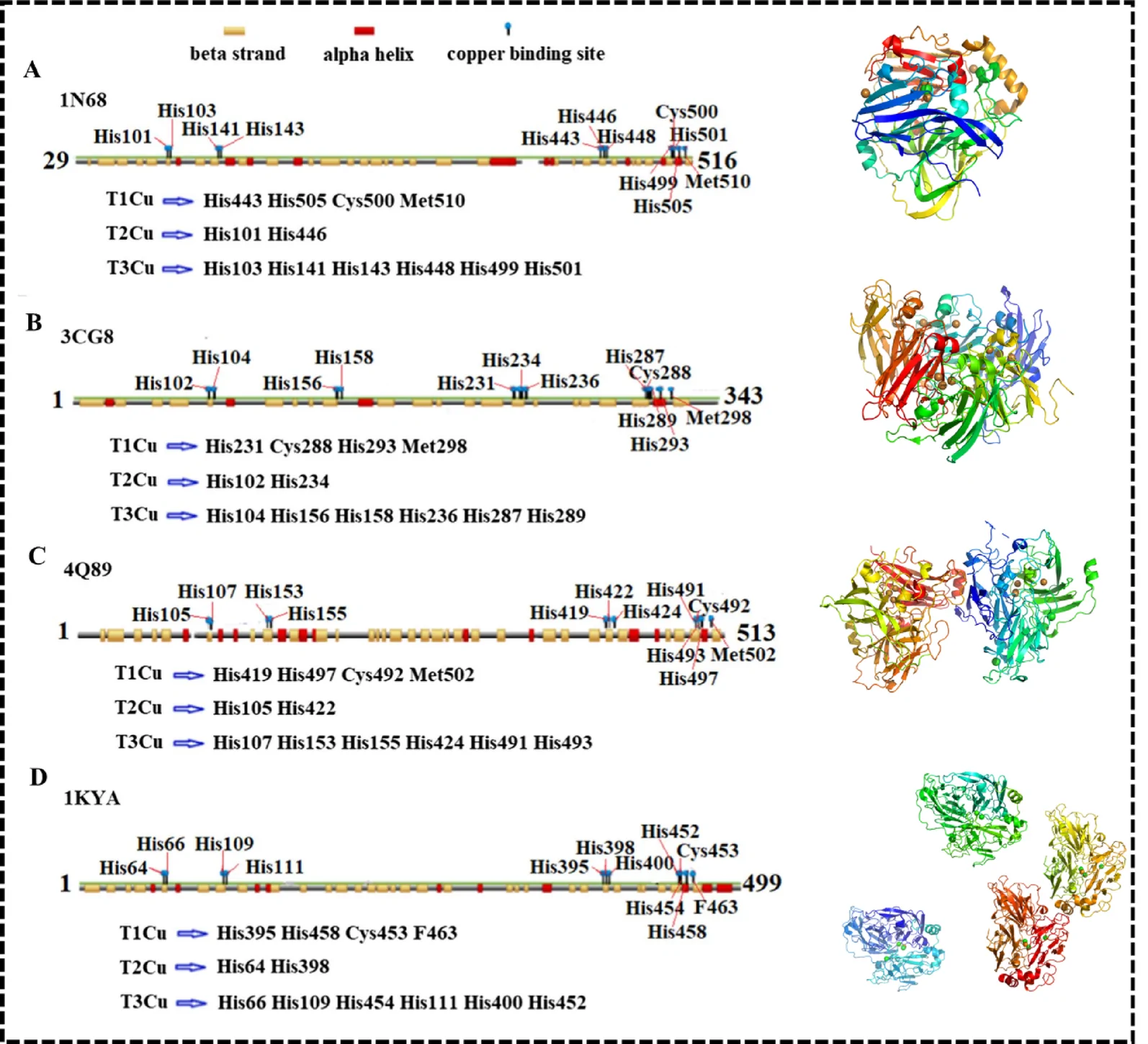
Secondary and tertiary structures and conserved copper-binding sites of four laccases. **A** *E. coli*, PDB: 1N68. **B** *S. coelicolor*, PDB: 3CG8. **C** *B. subtilis*, PDB: 4Q89. **D** *T. versicolor*, PDB: 1KYA (Guan et al. 2018)

The discovery of novel laccases has broadened the laccase pool for dye degradation, but the differences in decolorization performance among laccase sources need to be interpreted through defined biochemical and structural indicators. As illustrated in Fig. 5, bacterial and fungal laccases share the conserved multicopper oxidase architecture, yet differ in secondary- and tertiary-structure organisation, copper-binding microenvironments, surface properties and substrate-access channels, which can affect redox potential, substrate affinity and environmental tolerance (Guan et al. 2018). Fungal laccases are commonly secreted and glycosylated, favoring extracellular oxidation and high-redox-potential catalysis, whereas bacterial laccases are often non-glycosylated or weakly glycosylated and may display stronger tolerance to alkaline pH, temperature and salinity. These features partly explain variations in dye specificity, mediator dependence and decolorization efficiency. Table 2 summarizes representative laboratory-scale decolorization studies and provides a comparative basis for evaluating laccase performance in simulated dye-treatment systems. Table 2 was designed to enable comparison across multiple dimensions rather than ranking laccases solely according to reported decolorization percentages. The summarized parameters include enzyme origin, dye type, initial dye concentration, enzyme dosage, reaction temperature and pH, reaction duration, mediator supplementation and analytical approaches used for confirming dye transformation. Inclusion of these parameters allows differences in catalytic performance to be interpreted in the appropriate experimental context. To provide a broader perspective on the current development strategies for laccase-based dye treatment, a comparative summary of fungal laccases, bacterial laccases, recombinant enzymes, engineered variants and immobilized systems is presented in Table 3. These platforms represent different levels of technological development, ranging from natural enzyme discovery to molecular optimization and process-oriented catalyst design. Their advantages and limitations differ substantially in terms of catalytic performance, environmental tolerance, production feasibility, scalability and suitability for industrial wastewater treatment.

**Table 2:** Representative novel and recombinant laccases for dye decolorization and degradation

Source	Dye	Dye concentration	Mediator	Laccase amount	Decolorization	Conditions	References
*Trametes flavida* WTFP2	CR	100 mg/L	–	–	99.48%	16 h	Sharma et al. (2023)
*Gliomastix murorum*	CR EBT	200 mg/L	–	–	86.44% 75.98%	24 h; 25 °C	Fernández-Remacha et al. (2022)
*Alternaria* sp. D21	MO MB Acid Green 3 RBBR	100 mg/L	–	–	98.00% 78.00% 93.00% 71.00%	144 h; 30 °C	Toker et al. (2021)
*Trichoderma harzianum* AUMC14897	Light Green SF Yellowish CR MG	50 mg/L	–	20 U/mL	89% 77.89% 71.4%	pH 4.5; 50 °C; 24 h	Salem et al. (2024)
*Trichoderma asperellum* Tasjk65	MG CR MO CV Neutral Red	4.5 mg/L 300 mg/L 6 mg/L 4.5 mg/L 9 mg/L	–	–	73.0% 58.2% 35.6% 28.1% 25.9%	pH 4.8; 37 °C; 48 h	Fu et al. (2025)
*Bacillus safensis* HL3	IC	47 mg/L	AS (0.1 mM)	–	97.00%	2 h; 40 °C; pH 7.0	Wang et al. (2022a)
*Microcystis flos-aquae*	Cibacron Brilliant Blue	40 mg/L	–	–	78.00%	pH 4.0	Muhammad et al. (2024)
*Trametes trogii*	BF MG Direct Blue	50 mg/L	–	–	60.14% 95.10% 47.44%	10 h; 30 °C	Song et al. (2024)
*F. oxysporum* HUIB	RBBR Aniline Blue IC	10 mg/L	Vanaline (1 mM)	10 U	88.92% 91.63% 86.95%	–	Huy et al. (2021)
*Bacillus* sp. FNT	CR MO MR MG CV RBBR	50 mg/L	AS (2 mM)	0.1 mg/mL	Rapid decolorization of dyes	pH 6.0; 40 °C; 30 min	Espina et al. (2021)
*Yarrowia lipolytica*	EBT MO Bromophenol Blue	–	–	5 U/mL	62.30% 32.10% 46.30%	pH 5.0; 28 °C	Jiang et al. (2023)
*Anoxybacillus ayderensis* SK3-4	Direct Green 6 Direct Black 19	–	–	200 U/L	99.64% 51.34%	pH 7.5; 70 °C; 1 h	Wang et al. (2020)
–	MG	50 mg/L	–	–	> 95.00%	2 h	Lima et al. (2022)
*Catenuloplanes japonicus*	MO BG	50 µM	ABTS (50 µM)	200 mU	57.00% 94.00%	30 °C; 24 h; pH 4.0;	Trubitsina et al. (2021)
*Trametes hirsuta* MX2	RBBR	150 mg/L	–	0.05 U/mL	92.57%	pH 5.0; 3 h; 28 °C	Jia et al. (2022)
*Bacillus licheniformis* NS2324	MB	–	–	10 IU/mL	99.28%	40 °C; pH 8.0; 4 h	Chopra and Sondhi (2022)
*Pycnoporus sanguineus* MUCL 38,531	MG CV CR	100 µM 50 µM 50 µM	ABTS (100 µM)	0.1 U/mL	100.00% 90.00% 88.20%	30 °C; pH 4.0 for MG/CV and pH 6.0 for CR	Malcı et al. (2023)
*Methylobacterium extorquens*	CR IC	80 mg/L	–	1 U/mL	99.00% 54.00%	50 °C; pH 7.5 for CR and pH 1.5 for IC	Ainiwaer et al. (2022)
*Peniophora* sp. CBMAI 1063	IC	50 mg/L	SGZ (100 mM)	0.02 U/mL	93.00%	pH 5.0; 2 h; 30 °C	Otero et al. (2025)
*Bacillus amyloliquefaciens*	RB 5 IC	–	AS (0.1 mM)	50 U/L	93.00% 99.00%	pH 6.6; 40 °C; 1 h	Wang et al. (2021)
*Cerrena unicolor*	RBBR Indigo Blue	20 mg/L	–	0.2 U/mL	91.60% 87.10%	pH 4.5; 45 °C; 24 h	Huang et al. (2024)

**Table 3:** Comparative characteristics of fungal laccases, bacterial laccases, recombinant enzymes, engineered variants and immobilized laccase systems for dye wastewater treatment

Laccase platform	Main advantages	Main limitations	Typical application scenarios	Current industrial relevance	Future improvement strategies
Fungal laccases	High redox potential; broad substrate range; strong oxidation of aromatic dyes; extracellular secretion	Sensitive to pH, temperature and inhibitors; production cost; limited alkaline tolerance	Diverse aromatic dye degradation under mild conditions	Moderate; many studies remain laboratory-scale	Discovery of extremophilic fungi; protein engineering; improved fermentation
Bacterial laccases	High thermal, alkaline and salt tolerance; suitable for harsh wastewater conditions; easier genetic manipulation	Generally lower redox potential; narrower substrate range; weaker oxidation of high-potential dyes	Saline, alkaline and high-temperature dye wastewater	Increasing potential	Metagenomic discovery; recombinant expression; directed evolution
Recombinant laccases	Higher production yield; easier purification; suitable for engineering; scalable expression platforms	Expression bottlenecks; secretion efficiency; folding and glycosylation problems; host-dependent limitations	Production optimization and industrial enzyme manufacturing	Emerging	Improved hosts; secretion engineering; synthetic biology
Engineered variants	Tunable activity, stability, substrate specificity and mediator dependence; rational optimization possible	Requires screening/validation; possible trade-off between properties; limited real wastewater validation	Targeted dye degradation under specific conditions	Early-stage	AI-assisted design; machine learning; computational protein engineering
**I**mmobilized laccases	Improved stability, recovery, reuse and continuous operation potential	Carrier cost; enzyme leakage; diffusion limitation; possible nanomaterial concerns	Repeated batch and reactor-based wastewater treatment	Most promising for process implementation	Low-cost carriers; scalable immobilization; pilot-scale validation

Despite the advantages of recombinant laccase production, several challenges associated with heterologous expression remain major barriers for industrial implementation. One critical limitation is the requirement for appropriate post-translational modifications, particularly glycosylation, which can strongly influence enzyme folding, secretion efficiency, structural stability, and catalytic performance. As several fungal laccases are naturally glycosylated extracellular enzymes, recombinant expression in hosts with different glycosylation machinery may result in altered glycan patterns and reduced enzymatic activity or stability. In addition, efficient secretion of recombinant laccases remains challenging because high-level expression does not necessarily correspond to increased extracellular enzyme yield. Limitations associated with signal peptide recognition, intracellular accumulation, proteolytic degradation, and secretion pathway capacity may significantly reduce production efficiency. Proper protein folding and copper incorporation represent additional challenges, as incorrect folding or incomplete metal loading can generate inactive or poorly active recombinant enzymes. From an industrial perspective, recombinant laccase production must also consider economic factors, including host cultivation requirements, fermentation scale-up, downstream purification costs, and process optimization. Therefore, future development of recombinant laccases should focus not only on increasing catalytic activity but also on engineering expression systems that enable efficient secretion, correct post-translational modification, robust folding, and cost-effective large-scale enzyme production. Therefore, the practical value of recombinant laccases should be evaluated not only by expression yield, but also by their ability to efficiently oxidize structurally diverse dyes, maintain activity in the presence of wastewater inhibitors and provide a scalable enzyme source for continuous dye-treatment processes.

### Protein engineering

New laccase mining and recombinant expression provide laccases with diverse stability and catalytic profiles. Protein engineering extends this strategy by redesigning selected laccase scaffolds to strengthen specific properties that determine dye decolorization, including substrate affinity, mediator turnover, catalytic efficiency and tolerance to reaction conditions (Barber-Zucker et al. 2022). Importantly, the success of protein engineering in laccase-based dye treatment should be assessed according to its impact on dye degradation rather than enzyme parameters alone. Improvements in catalytic efficiency, substrate affinity or stability are meaningful only when they translate into faster decolorization, broader dye specificity, reduced mediator dependence or improved performance in complex wastewater matrices. For dye wastewater treatment, immobilization should therefore be considered not merely as an enzyme stabilization approach, while as a method for converting laccase into a reusable catalytic platform. An effective immobilization system must simultaneously preserve the catalytic architecture required for dye oxidation, maintain accessibility of dye molecules to the active site and enable repeated operation under realistic wastewater conditions. Rational design, directed evolution and semi-rational mutagenesis have therefore been used to generate variants with quantifiable improvements in activity, stability or dye-removal rate (Mate and Alcalde 2015; Dabirmanesh et al. 2015). Although these three protein engineering strategies share the common goal of improving laccase performance, they differ substantially in their design principles, screening requirements, and practical applicability. Rational design relies on structural and mechanistic information to introduce targeted mutations at functionally relevant residues, providing high interpretability and efficient optimization of specific properties, while its success is limited by incomplete understanding of structure–function relationships. Directed evolution generates extensive sequence diversity through random mutagenesis and high-throughput screening, enabling the discovery of beneficial variants without prior structural knowledge; however, it generally requires notable screening capacity and may provide limited mechanistic insight. Semi-rational engineering integrates the advantages of both approaches by combining structure-guided selection of mutation sites with concentrated library construction, thereby reducing screening requirements while maintaining the possibility of obtaining significant functional improvements. In practical laccase applications, rational design is particularly suitable for modifying known limitations, such as substrate accessibility or thermal stability, directed evolution is advantageous for improving complex traits under application-relevant selection pressures, and semi-rational approaches represent a balanced strategy for tailoring laccases toward specific dye substrates or wastewater conditions.

Importantly, improving laccase catalytic performance requires consideration of substrate-specific structural constraints. Mutations introduced through protein engineering may enhance substrate binding by enlarging active-site accessibility, modifying local charge distribution, or improving interactions with aromatic substrates. Therefore, enzyme engineering strategies should not only aim to increase intrinsic catalytic activity or redox potential but also optimize molecular recognition between engineered laccases and structurally diverse dye molecules.

#### Rational design

Rational design provides a structure-guided route to improve laccase-mediated dye decolorization by modifying residues that determine how dyes or redox mediators approach and react at the T1 Cu (Pardo and Camarero 2015). Unlike enzyme mining, rational engineering starts from a known enzyme and targets the structural constraints that limit dye removal, such as weak substrate or mediator binding, restricted access of bulky chromophores, insufficient catalytic efficiency or poor stability under decolorization conditions (Raen et al. 2025). Recent studies have therefore used structural modelling, molecular docking and targeted mutagenesis to improve dye-decolorizing performance, with gains commonly reflected in higher *k*_cat_/*K*_m_, increased specific activity, longer half-life, improved residual activity or faster dye removal (Mate and Alcalde 2015). These improvements are valuable because they connect molecular modification with measurable decolorization outcomes, although most examples still require further validation in mixed-dye or real wastewater systems. Therefore, enhanced catalytic activity observed after molecular engineering should be interpreted as an improvement in intrinsic enzyme performance rather than direct evidence of industrial applicability. Comprehensive evaluation of engineered laccases should additionally consider substrate diversity, resistance to wastewater-associated inhibitors, operational stability, scalability of enzyme production, and compatibility with reactor-based treatment processes.

**Fig. 6:**
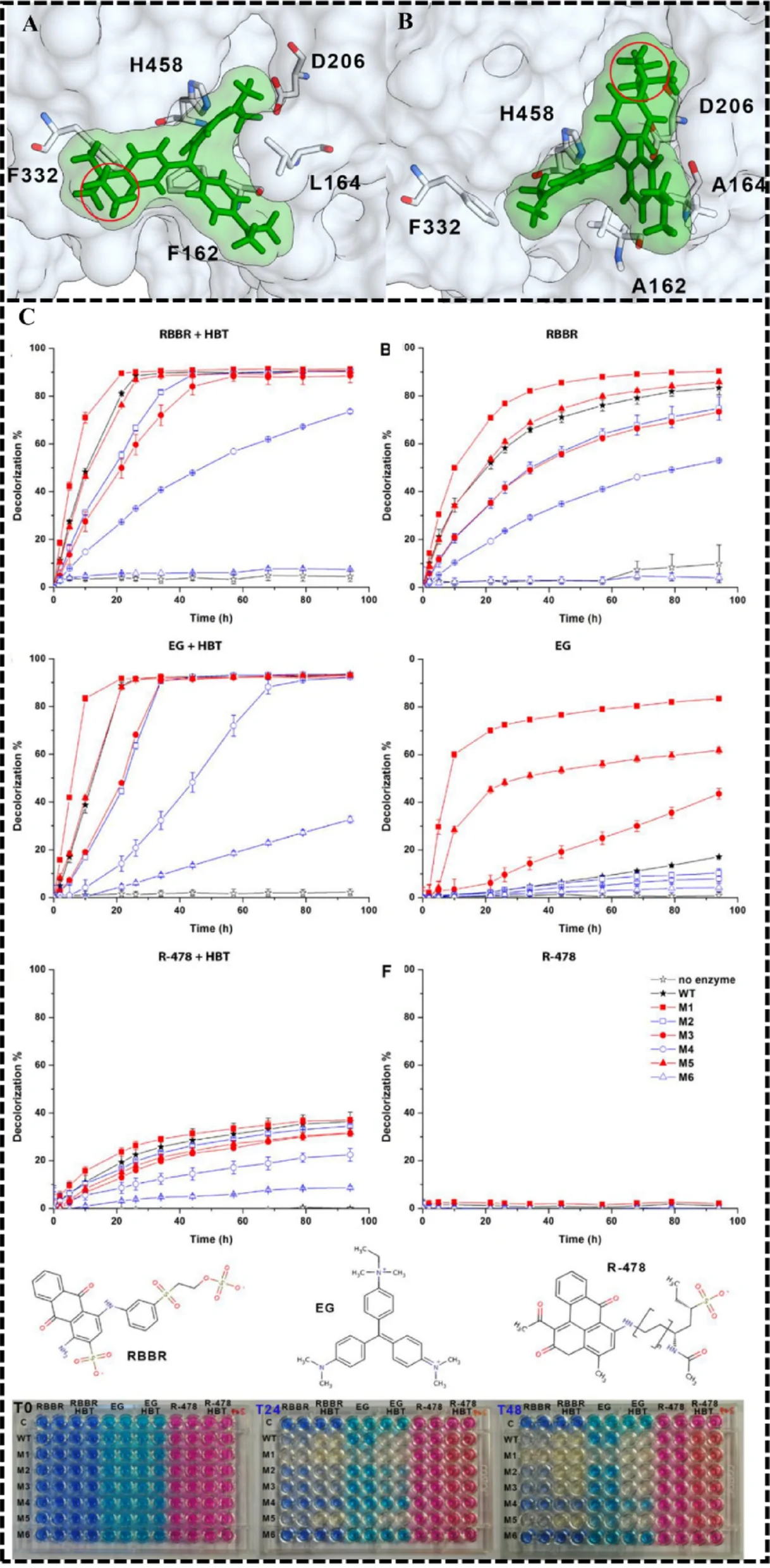
EG docking and dye decolorization by WT and M1 laccases. **A**, **B** Top-ranked EG docking poses with WT and M1; the ethyl moiety is circled in red. **C** Decolorization of RBBR, EG and R 478 at pH 5.0 with or without HBT, with representative 96-well plate images (Chiadò et al. 2021)

Active-site pocket engineering has yielded some of the most direct quantitative gains in laccase-mediated dye decolorization (Kolyadenko et al. 2021). For the lccβ laccase from *T. versicolor*, F162A/L164A substitutions enlarged the substrate-binding pocket, increased ABTS activity to approximately 300% of the WT level at pH 3.0, and enabled 72.5% mediator-free Ethyl Green (EG) decolorization (Fig. 6), whereas the WT showed little activity under the same conditions (Chiadò et al. 2021). The main significance is not only the increase in model-substrate activity, but the conversion of a nearly inactive enzyme–dye combination into a productive reaction by reshaping substrate access within the catalytic pocket. Thus, pocket remodelling should be interpreted as a substrate-selective strategy: it is most effective when poor decolorization arises from steric mismatch between bulky chromophores and the active-site entrance.

Residues outside the immediate substrate-binding pocket can also alter dye-removal profiles (Kunamneni et al. 2008). In the two-domain laccase ScaSL, H286 is located between the T1 and TNC, whereas F232 and F233 are positioned in the second coordination sphere of the T1 Cu. H286T increased the redox potential by 0.51 V, while F232Y/F233Y improved ABTS-assisted CR decolorization but abolished direct decolorization of hydrophobic triphenylmethane dyes (Trubitsina et al. 2024). This contrast highlights a recurrent limitation of targeted mutagenesis: local changes that favor one dye class may narrow the substrate spectrum for another. In *B. subtilis* CotA, Thr466 is located near the water-channel region close to the TNC; its substitution by alanine increased Reactive Blue 19 (RB 19) and EBT decolorization to 42.24% and 58.20% within 1 h, compared with 14.17% and 36.15% for the WT, respectively (Zhou et al. 2025). Such distal mutations are less intuitive than active-pocket substitutions, but they broaden the design space by linking dye removal to residues involved in the wider catalytic cycle.

Stability-oriented rational design provides a complementary route when dye removal is limited by reaction temperature or pH. In Ghlac, Mut2 increased the half-life at 60 °C from 3.6 to 9.8 h and improved MG decolorization at 70 °C from 78% for the WT to complete decolorization within 3 h (Mao et al. 2021). LacCT-11 (G190P/Q254Y/G336M/D510F) further illustrates this strategy: loop rigidification and strengthened hydrophobic interactions extended the half-life at 60 °C by 1.8-fold, while Reactive Black B and Reactive Black WNN decolorization reached 88% and 86%, respectively, at pH 7.5 and 50 °C (Xu et al. 2024). These stability gains are particularly relevant for textile-related reaction conditions, but they remain largely demonstrated in defined dye systems rather than real effluent matrices.

Rational design enables targeted improvement of laccase-mediated dye decolorization by linking structural modification to measurable gains in activity, stability or dye-removal rate. Rational design is expected to overcome the bottlenecks of traditional protein engineering in the future, enabling the predictable design and efficient development of laccase functions.

#### Directed evolution

Directed evolution complements rational design by generating sequence diversity through random mutagenesis or recombination and selecting variants with improved activity, stability or substrate adaptability, without requiring detailed structural or mechanistic information (Wang et al. 2012). Laccases are suitable targets because their multicopper scaffold tolerates sequence variation and their broad substrate range enables functional screening for dye-related phenotypes (Stanzione et al. 2020).

Recent studies have linked screening design more directly with dye-removal performance. Using MG as the selection pressure, Li et al. (2025b) evolved *Bacillus pumilus* CotA and obtained PW4G, whose specific activity increased from 33.22 to 100.66 U/mg and whose catalytic efficiency towards ABTS increased 6.08-fold. Under pH 9.0 and 50 °C, PW2 and PW4G decolorized more than 96% of MG within 5 h in the presence of ABTS, approximately 25% higher than the WT. This dye-pressure strategy is valuable because the screening condition is aligned with the target pollutant, allowing variants with improved MG tolerance, expression-associated growth advantage and dye-removal capacity to be enriched in the same workflow. The improvement was less pronounced for CR and X-BR, indicating that dye-pressure screening can strongly favor the selection dye but may not generate broad-spectrum decolorization.

One important application of directed evolution in fungal laccases is to improve activity under neutral or alkaline conditions, where many native fungal enzymes perform poorly. Yin et al. (2019) evolved Coprinopsis cinerea Lcc9 into PIE5 (E116K/N229D/I393T), shifting the optimum pH for guaiacol oxidation to 8.5. At 60 °C, PIE5 decolorized 200 µM indigo dye by 90.9% within 3 h with 200 µM ABTS at pH 7.0, compared with 71.9% and 75.8% for wLcc9 and rLcc9, respectively. With 120 µM methyl 3,5-dimethoxy-4-hydroxybenzoate, PIE5 achieved 87.1% decolorization within 50 min at pH 7.5. However, efficient decolorization remained mediator-dependent, and PIE5 showed lower thermal and pH stability than the parental enzyme. The main advance of PIE5 is therefore not simply higher dye removal, but the relocation of fungal laccase activity into a neutral-to-alkaline operating window; the accompanying dependence on mediators and reduced thermal and pH stability also show that pH-profile engineering may redistribute, rather than uniformly improve, enzyme performance. Other fungal engineering examples, such as the V159E/N398D variant of *Agrocybe pediades* laccase, further improved activity near pH 6.0 and oxidation of Evans Blue (EB) and Reactive Black 5 (RB 5) (Aza et al. 2021). Directed evolution can also target whole-cell performance. Jeon et al. (2025) obtained copper-resistant *Bacillus velezensis* mutants with up to 2.6-fold higher laccase activity under copper stress and improved CR decolorization from 40.79% to 76.55% within 6 h. This strategy is relevant to copper-containing dye systems, but the gain is likely linked to host-level copper uptake, homeostasis and laccase maturation rather than direct optimization of the laccase protein.

Directed evolution provides an effective strategy for improving laccase-mediated dye decolorization, especially when screening conditions reflect the intended treatment environment. Although reported improvements often depend on the target dye, mediator or host, this specificity can guide laccase tailoring for defined wastewater conditions. Future studies should therefore link evolutionary screening more closely with mixed-dye systems and real-effluent validation.

#### Semi-rational design

**Fig. 7:**
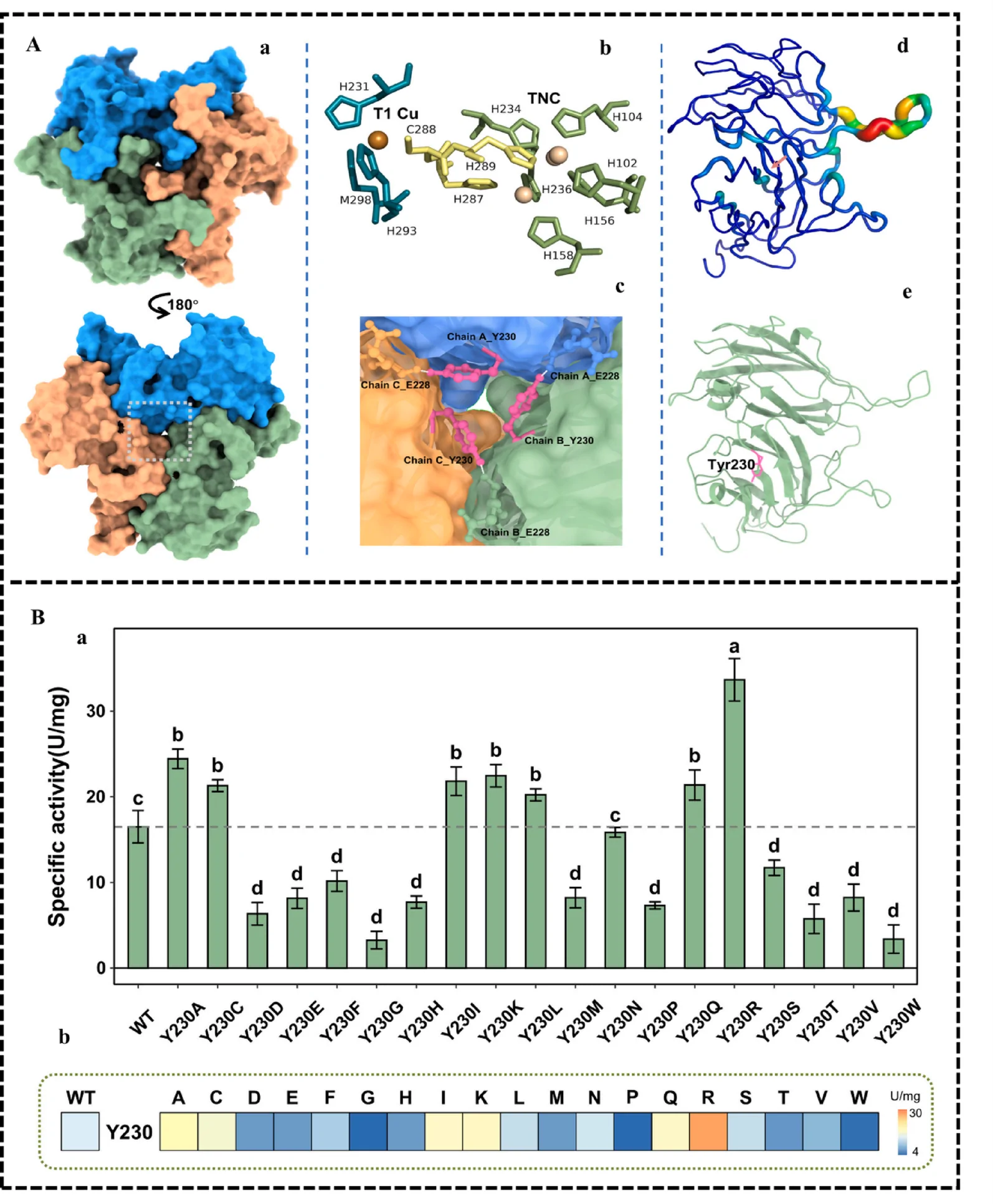
Structure-guided Y230 mutation and activity of SLAC. **A** SLAC structural model based on PDB 3CG8, showing the catalytic centre, Y230 location and deformation energy. **B** Specific activities of WT and Y230 mutants and heatmap of mutant activities relative to WT (Bian et al. 2024b)

Semi-rational design combines the targeting precision of rational design, with the high-throughput screening capability of directed evolution (Chica et al. 2005). It enables the focused introduction of diversity at key residues to optimize laccase properties with controlled mutation scope and fewer screening iterations (Lutz 2010). In the context of semi-rational laccase design, saturation mutagenesis plays a key role in functional optimization. Recent work on *Streptomyces coelicolor* SLAC illustrates this point. As shown in Fig. 7, Tyr230 is positioned near the T1 Cu-centerd substrate-access region and adjacent to E228 at the inter-chain interface. Saturation mutagenesis identified Y230R as the most active variant, increasing specific activity from 16.5 to 33.7 U/mg and catalytic efficiency towards ABTS from 1.31 to 1.64 s⁻¹ mM⁻¹ (Bian et al. 2024b). Y230R also retained more than 80% relative activity at pH 5.0 and improved MG decolorization at pH 6.0 from 61.1% to 67.0%. Engineering the adjacent E228 position revealed that improvements in ABTS oxidation and MG decolorization do not necessarily follow the same trend: E228F markedly increased ABTS affinity, whereas E228G produced the highest MG decolorization, increasing removal from 54% to 67% (Bian et al. 2024a). Dye decolorization is not determined solely by model-substrate kinetics, but also by how mutations reshape substrate-pocket access for structurally distinct dye molecules, providing a reference for target-dye-guided substrate-pocket engineering.

CotA-laccase engineering provides a clearer case for mediator-free azo-dye decolorization under alkaline conditions. In *Bacillus pumilus* W3 CotA, Ma et al. (2020) used docking-guided saturation mutagenesis at Thr415, Thr418 and Gln442 to improve RB 5 decolorization. The T418K/Q442A mutant increased catalytic efficiency towards ABTS by 5.33-fold and towards RB 5 from 6.99 to 22.54 s⁻¹ mM⁻¹, and decolorized 52.64% RB 5 at pH 10.0 and 37 °C within 24 h without a mediator, compared with 11.88% for the WT. This result indicates that steric accommodation at the substrate-binding entrance is a decisive constraint in mediator-free RB 5 oxidation. For bulky azo dyes, gains in decolorization require both efficient electron transfer near the T1 Cu region and productive substrate access. A second CotA variant, T377I/T418G, extended this strategy to CR, achieving 76.59% mediator-free decolorization at pH 8.0 and 50 °C, with catalytic efficiency towards CR increasing from 1.79 to 3.96 s⁻¹ mM⁻¹ (Yan et al. 2022). By linking improved catalytic efficiency with substrate-pocket accessibility, these CotA variants demonstrate how semi-rational design can reduce mediator dependence in alkaline azo-dye decolorization. Complementary semi-rational studies extend this design logic from substrate-pocket access to operational stability and intramolecular electron transfer. CtLac V243D improved oxidase activity under thermoalkaline conditions, whereas CueO D360K enhanced bioelectrocatalytic current by favoring electron transfer between the fifth copper and the T1 Cu (Zhang et al. 2020a; Yang et al. 2023c). The examples above collectively show that protein engineering provides a route to convert naturally dye-active laccases into catalysts with more defined decolorization performance. By reshaping catalytic residues, substrate-access pathways and stability-related regions, rational design, directed evolution and semi-rational mutagenesis allow laccase properties to be tuned towards specific dye structures and treatment conditions. This shift from enzyme discovery to targeted optimization is central to developing laccases that are better suited for dye-contaminated wastewater.

Recently, artificial intelligence (AI)-assisted protein engineering has emerged as a complementary strategy for accelerating laccase optimization beyond conventional structure-guided and screening-based approaches. In contrast to traditional rational design, which depends on limited structural information, AI models can integrate large-scale sequence, structural and functional datasets to identify sequence patterns associated with catalytic activity, substrate specificity and environmental stability. Machine learning algorithms, protein language models and structure-prediction tools can assist in prioritizing beneficial mutations, predicting epistatic interactions and reducing the experimental burden associated with screening large mutant libraries. For laccases, AI-guided approaches may promote the redesign of substrate-binding pockets, improvement of redox properties, enhancement of thermal or pH stability, and development of variants with reduced mediator dependence.

While protein engineering enables the intrinsic optimization of laccase catalytic properties, immobilization engineering provides an external regulatory environment that determines enzyme accessibility, stability, recovery, and long-term operation. Consequently, effective industrial application of engineered laccases requires simultaneous consideration of both molecular modification and carrier-assisted stabilization strategies.

## Materials for immobilized laccase-mediated dye decolorization

Immobilization strategies differ substantially in their effects on enzyme performance, and the selection of an appropriate immobilization method requires balancing catalytic activity with operational stability and process feasibility. Physical adsorption approaches generally provide simple and low-cost enzyme immobilization with relatively mild preparation conditions; however, weak interactions between enzyme and carrier may result in enzyme leakage and reduced long-term stability. Covalent immobilization can significantly improve enzyme retention and operational lifetime by forming stable enzyme–support interactions, although excessive modification of surface residues may reduce catalytic activity by restricting conformational flexibility or blocking substrate-accessible regions. Entrapment and encapsulation strategies can protect enzymes from environmental stress and minimize leakage, but diffusion limitations may decrease apparent activity, particularly for large dye molecules. From an industrial perspective, immobilization performance should not be evaluated solely based on initial enzyme loading or decolorization efficiency. Important parameters include retained catalytic activity after immobilization, resistance to temperature and pH fluctuations, number of reuse cycles, enzyme leakage during operation, long-term operational lifetime, carrier cost, scalability, and compatibility with continuous wastewater treatment systems. Therefore, the optimal immobilization strategy depends on the intended application conditions and requires simultaneous consideration of catalytic efficiency, durability, and economic feasibility.

Free laccases can catalyse the oxidation of structurally diverse dyes, but their practical use is often limited by enzyme inactivation, poor recovery and single-use operation (Datta et al. 2021). Immobilization provides a material-based strategy to address these limitations by placing laccase within a defined carrier microenvironment (Wang et al. 2024). In this context, the support is not merely a physical holder for the enzyme; it regulates enzyme conformation, substrate accessibility, local dye concentration and catalyst recovery, all of which influence decolorization performance. For engineered laccases, immobilization is particularly important because introduced mutations may improve catalytic properties while simultaneously affecting folding stability, surface charge distribution, or interaction with support materials. Therefore, carrier selection should be tailored not only to the native enzyme characteristics but also to the structural and functional properties of engineered variants. From an industrial translation perspective, the most promising immobilization platforms are unlikely to be those exhibiting the highest activity enhancement under optimized laboratory conditions, but rather those capable of maintaining stable enzyme performance during long-term operation in complex wastewater environments. Consequently, carrier selection should increasingly focus on continuous-flow compatibility, low-cost manufacturing, environmental safety, and practical recovery strategies. For practical wastewater treatment applications, kinetic improvements observed under ideal laboratory conditions should be interpreted together with mass-transfer behavior and long-term operational stability. An immobilized laccase system with high enzyme loading does not necessarily provide superior treatment performance if substrate diffusion is restricted or active sites become inaccessible during repeated operation. Therefore, future immobilization design should optimize not only enzyme retention, but also the balance between catalytic activity, transport efficiency, and process scalability.

The immobilization mode determines how laccase is retained and how dye molecules approach the catalytic site (Prabhakar et al. 2025). Physical adsorption relies on van der Waals forces, ionic bonding, and hydrophobic effects (Pandey et al. 2020). While this method is easy to operate and has minimal effect on laccase activity, the weak binding can lead to laccase leakage. Covalent binding, which involves the formation of stable covalent bonds between the laccase and carrier functional groups (such as amino and carboxyl groups), circumvents such leakage. Nevertheless, it may cause damage to the laccase active site due to chemical modification (Madhu and Chakraborty 2017). Entrapment immobilises laccase by confining it within a porous matrix or semi-permeable membrane, effectively shielding the enzyme from external interference. However, mass transfer limitations frequently affect the diffusion efficiency of the substrate (Wong et al. 2019). Cross-linking involves the connection of laccase molecules with cross-linking agents such as glutaraldehyde to form a 3D network structure, thereby enhancing stability. However, this may result in laccase inactivation due to aggregation (Chen et al. 2025). These differences explain why immobilized laccases with similar enzyme sources can show markedly different decolorization efficiencies and reuse behaviors.

Carrier properties further shape the performance of immobilized laccase systems. Surface functional groups affect enzyme loading and orientation; porosity controls mass transfer; adsorption capacity can enrich dye molecules near the enzyme; and mechanical or magnetic properties facilitate catalyst recovery (Alvarado-Ramírez et al. 2021). The support therefore determines not only dye removal, but also activity retention, enzyme leakage, mass transfer and repeated-use performance. Current immobilization materials, including carbon-based nanomaterials (CNMs), metal-based materials, porous frameworks, biopolymer composites, clay minerals and macroscopic carriers, improve laccase-mediated dye decolorization through different combinations of stabilization, adsorption, electron transfer and recyclability (Wang et al. 2023; Patil et al. 2025).

Beyond improving operational stability, immobilization can remarkably alter the kinetic behavior of laccase by modifying enzyme conformation, orientation, substrate accessibility, and local microenvironmental conditions. Compared with free enzymes, immobilized laccases often exhibit changes in apparent Michaelis constant (*K*_m_) and maximum reaction velocity (*V*_max_). An increased apparent *K*_m_ generally reflects reduced substrate accessibility caused by steric constraints, stronger enzyme–carrier interactions, or diffusion limitations within the immobilization matrix, whereas a decreased *K*_m_ may indicate enhanced substrate enrichment or favorable orientation of the enzyme on the carrier surface. Similarly, Vmax may decrease after immobilization due to restricted conformational flexibility or limited substrate transport, although certain carriers can preserve or even improve catalytic performance by stabilizing the active conformation of the enzyme. The overall catalytic efficiency of immobilized laccase is therefore determined by the balance between enzyme stabilization and mass-transfer resistance. Porous carriers with optimized pore size and surface chemistry can facilitate substrate diffusion while maintaining enzyme accessibility, whereas excessive confinement may create diffusion barriers, particularly for large dye molecules with complex aromatic structures. Consequently, evaluation of immobilized laccase systems should include not only activity retention and reusability but also kinetic parameters (*K*_m_, *V*_max_, and catalytic efficiency), substrate diffusion characteristics, and enzyme–carrier interactions to better predict performance under realistic wastewater treatment conditions.

Although numerous immobilization carriers have demonstrated improved laccase stability and reusability at the laboratory scale, their suitability for large-scale wastewater treatment depends on multiple factors beyond catalytic performance, including material cost, preparation complexity, scalability, mechanical stability, regeneration capability, environmental compatibility, and long-term operational reliability. Among currently investigated materials, magnetic carriers and naturally derived polymeric supports such as chitosan represent some of the most practically promising candidates because they combine relatively simple preparation, acceptable biocompatibility, and improved enzyme recovery through magnetic separation or mild regeneration processes. Nanostructured materials, including graphene-based materials and metal–organic frameworks (MOFs), provide exceptional surface areas, tunable interactions, and enhanced enzyme stabilization; however, their large-scale implementation remains constrained by production costs, potential environmental concerns associated with nanomaterial release, and challenges in reproducible large-scale synthesis. In contrast, low-cost and biodegradable carriers may offer greater industrial feasibility despite sometimes providing lower enzyme loading or catalytic enhancement. Therefore, future development should prioritize not only maximizing immobilized laccase activity, but also achieving a balance among catalytic performance, material sustainability, process scalability, and economic viability.

### CNMs

CNMs are widely used as laccase supports because they combine high surface area, tunable surface chemistry and strong adsorption affinity towards aromatic dye molecules (Peng et al. 2020). Graphene-based materials, carbon nanotubes (CNTs), biochar and fullerenes can alter dye removal by changing enzyme loading, substrate diffusion, local dye enrichment, electron transfer and catalyst reuse (Nishshankage et al. 2024).

**Fig. 8:**
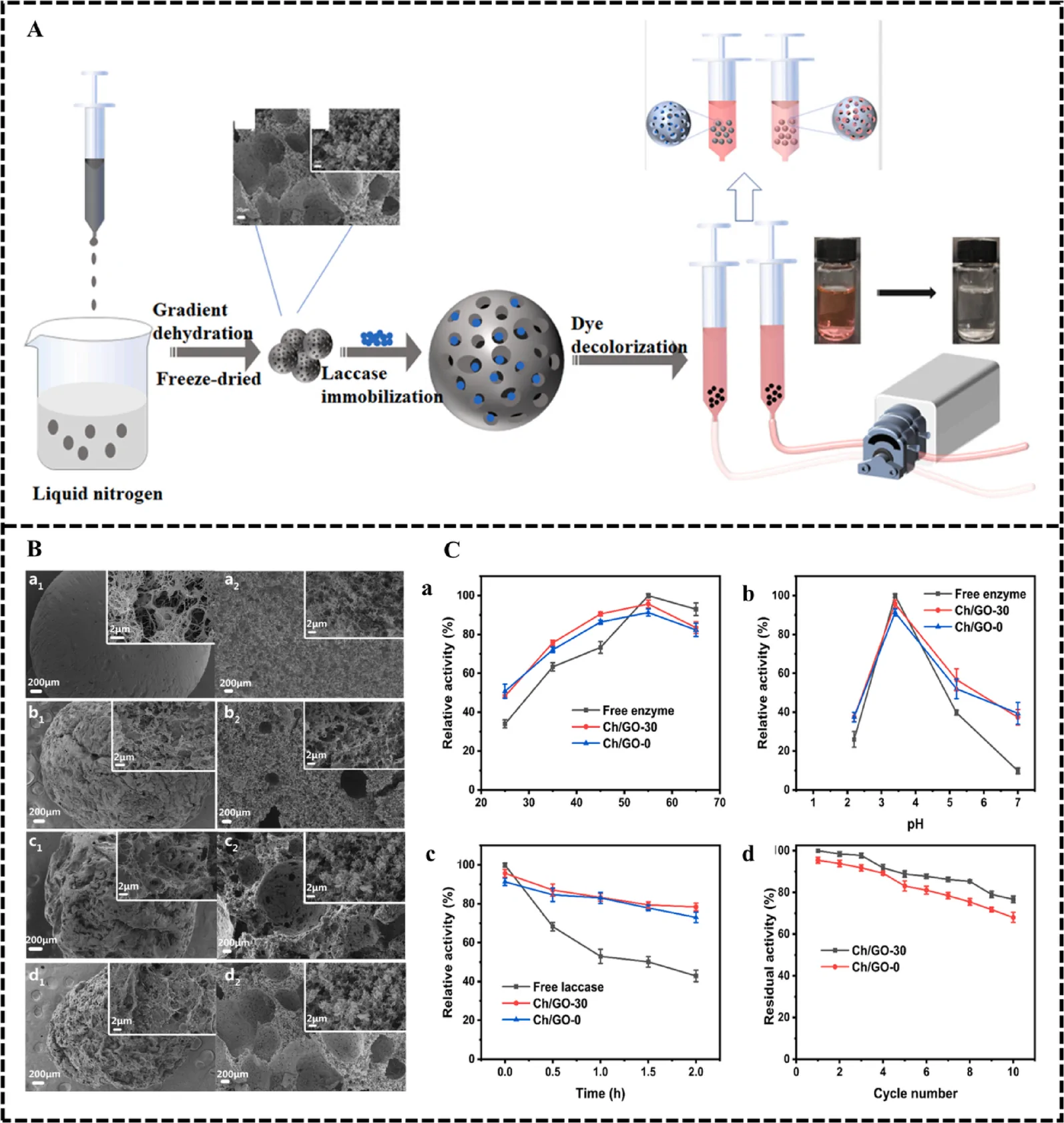
Immobilisation of laccase on Ch/GO composites and catalytic performance. **A** Schematic illustration of laccase immobilisation and decolorization. **B** SEM images of Ch/GO composites with different GO contents. **C** Effect of temperature and pH on enzyme activity, thermal stability and reusability of immobilized laccase (Huang et al. 2023)

GO-based supports illustrate the advantage of functionalized surfaces for enzyme loading (Adeel et al. 2018). Functionalized GO immobilized with *T. versicolor* laccase achieved 30.0 mg/g laccase loading and 96.87% relative activity at optimal loading, while decolorizing textile effluents by 71.25–88.65% (Noreen et al. 2021). Its kinetic improvement, reflected by lower *K*_m_ and higher *V*_max_ towards ABTS, suggests stronger enzyme–support interaction and improved substrate accessibility. The limitation appears during reuse: activity declined from 84.25% after two cycles to 54.47% after six cycles and to more than 40% after seven cycles, consistent with gradual enzyme leakage, aggregation or loss of enzyme–support compatibility. Hierarchical chitin/GO porous beads improved this repeated-use profile (Fig. 8), retaining 77% activity after 10 cycles and achieving near-complete decolorization of Acid Brilliant Scarlet GR and RB 19 (Huang et al. 2023). However, the same porous structure that improves mass transfer also enhances dye adsorption.

**Fig. 9:**
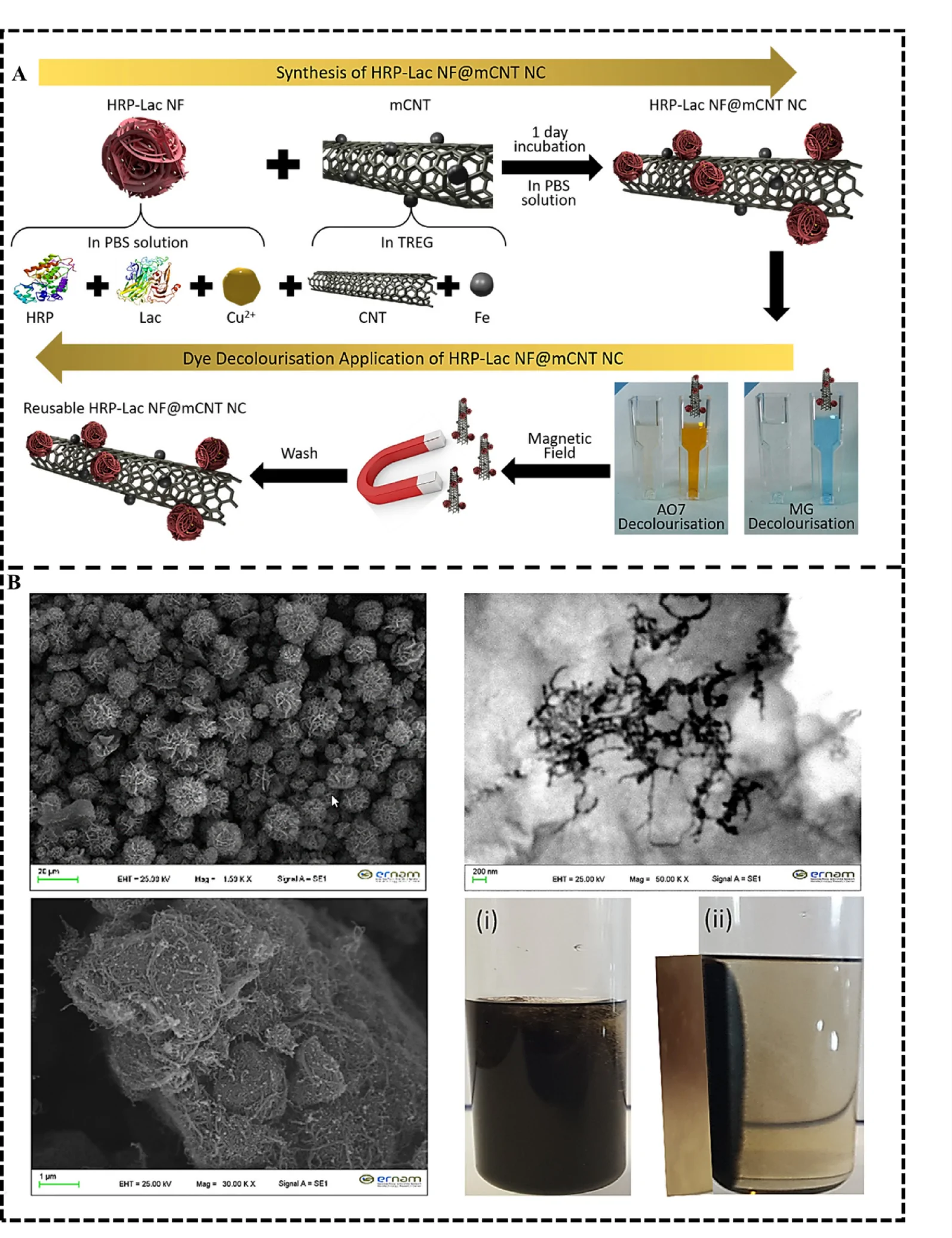
Synthesis and characterisation of HRP-Lac NF@mCNT nanocomposite for dye decolorization. **A** Schematic illustration of HRP-Lac NF@mCNT synthesis and magnetically recoverable dye decolorization. **B** SEM images of HRP-Lac NFs, mCNT and HRP-Lac NF@mCNT, with magnetic response of the nanocomposite (Gul and Ocsoy 2021)

CNT-based systems are most useful when adsorption sites are regenerated by immobilized laccase (Mazari et al. 2021). Laccase-CNTs increased CR removal from 84% for pristine CNTs to 96%, while increasing adsorption capacity from 625 to 769.23 mg/g (Zhang et al. 2020b). This improvement reflects a coupled process in which adsorbed dye molecules are oxidized by laccase, delaying adsorbent saturation. Magnetic CNT composites improve recovery and repeated use. HRP-Lac nanoflower@mCNT maintained high MG and Acid Orange 7 (AO 7) decolorization after 16 cycles (Fig. 9) (Gul and Ocsoy 2021), whereas laccase@Fe_3_O_4_-MWCNT@SiO_2_ retained 87% activity after 10 ABTS oxidation cycles and decolorized Acid Red 88, EBT and RB 5 by 98%, 99% and 66%, respectively, without a mediator (Habimana et al. 2021). Magnetic recovery strengthens recyclability, but the lower RB 5 removal shows that CNT-based immobilization remains constrained by dye structure.

Biochar-based supports provide a low-cost carbon platform in which dye adsorption and enzyme immobilization occur on the same material surface (Nishshankage et al. 2024). Alkali-modified rice-straw biochar showed a high MG adsorption capacity of 757.58 mg/g, and laccase immobilization increased MG removal from 89.68% with free laccase to 97.70–98.77%, depending on the immobilization mode (Wang et al. 2022b). LC–MS analysis further identified demethylated and hydroxylated products, supporting dye transformation rather than color removal alone. Bamboo biochar adds an important design criterion: immobilized laccase activity was governed mainly by surface carboxyl groups rather than specific surface area. Under optimized preparation conditions, the laccase/biochar composite reached 1404.17 U/g, retained 70.98% activity after 10 cycles, and removed 85.88% MG and 94.53% MO without added mediators (Zhang et al. 2025). Biochar systems are therefore attractive because they combine adsorption, enzyme stabilization and surface functionalisation, but their high adsorption capacity also means that decolorization data should be interpreted together with degradation-product or toxicity evidence. Fullerene-based supports remain a more exploratory branch of CNM immobilization. Oxidized C_60_ derivatives improved laccase dispersibility and enabled rapid HBT-assisted decolorization of Coomassie Brilliant Blue and Bromophenol Blue, but their limited dye range and mediator dependence make them less mature than GO, CNT or biochar supports for dye decolorization (Zygouri et al. 2020).

### Metal-based nanomaterials and metal–organic frameworks

Metal-based nanomaterials and metal–organic frameworks have expanded the design of immobilized laccase systems from simple enzyme attachment to interfacial regulation (Ovid’ko et al. 2018). Through tunable surface chemistry, metal coordination and porous architectures, these carriers can influence enzyme stability, substrate access, electron transfer and catalyst recovery (Xia et al. 2023).

Copper-based nanomaterials are mechanistically attractive for laccase systems because laccases are multicopper oxidases and their catalytic cycle depends on intramolecular electron transfer among copper centers (Suarez et al. 2025). Sridharan et al. (2021) prepared flaky triangular CuI nanoparticles and combined them with crude bacterial laccase for azo-dye degradation. Compared with free laccase, which showed limited decolorization over 72 h, the CuI/laccase system markedly accelerated Reactive Brown and Reactive Black removal within 60 min. The improvement probably reflects a combined contribution from enzymatic oxidation and CuI-mediated adsorption or photocatalytic reactions.

Magnetic metal-based supports are most useful when catalyst recovery, repeated use and storage stability are prioritised. Magnetic MOFs provide a representative example, with laccase@MMOFs retaining up to 89% residual activity after 10 reuse cycles and showing only slight activity loss after 30 days of storage (Ladole et al. 2020). This performance suggests that magnetic MOFs combine convenient magnetic recovery with framework-assisted enzyme protection, thereby improving the operational durability of immobilized laccase. However, mechanistic attribution should be made cautiously because dye removal may arise not only from immobilized laccase catalysis but also from support-associated adsorption or peroxidase-mimicking activity.

**Fig. 10:**
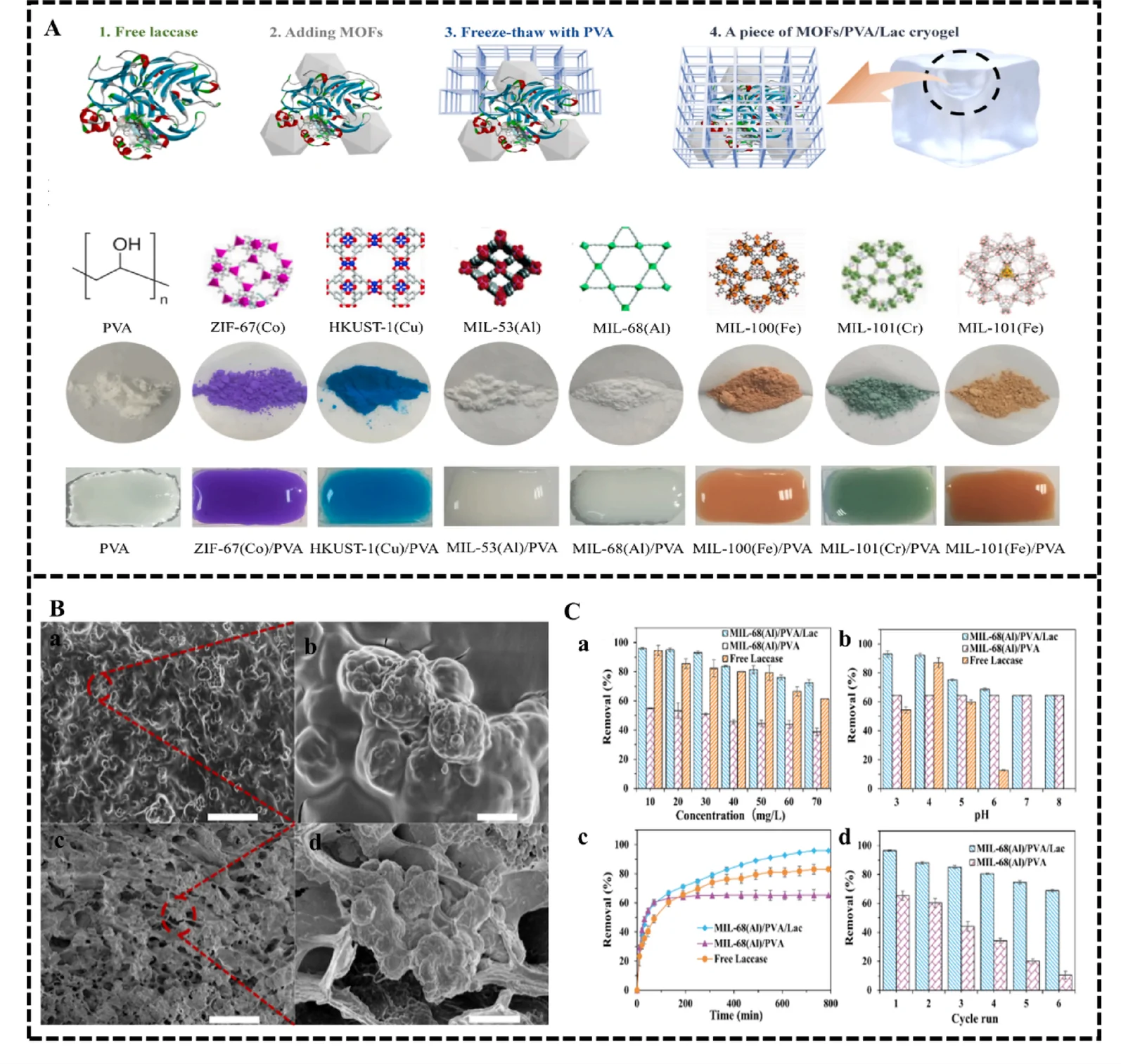
MIL-68(Al)/PVA/Lac cryogel for AG removal. **A** Preparation of MOF/PVA/Lac cryogels and representative samples. **B** SEM images of freeze-dried surfaces and cross-sections. **C** Effects of dye concentration, pH, time and reuse cycles on removal efficiency, with MIL-68(Al)/PVA and free laccase as controls where applicable (Peng et al. 2021)

Beyond redox assistance and magnetic recovery, MOF-based supports provide a microenvironmental strategy for laccase immobilization (Han et al. 2022). Their porous frameworks can protect laccase, retain mediators or enrich dye molecules near catalytic sites, but these benefits depend strongly on framework composition and mass-transfer behavior (Huang et al. 2022). MIL-68(Al)/PVA/Lac removed 95.86% of AG within 12 h and retained more than 60% removal after six cycles, with MS, 2D-NMR and docking supporting C-α oxidation rather than color loss alone (Fig. 10) (Peng et al. 2021). However, the increased *K*_m_ and decreased *V*_max_ after immobilization indicate that improved stability was accompanied by diffusion resistance. PET/UiO-66(Zr)-NH_2_/Lac/ABTS improved CV removal from 8.61% to 58.80% by co-localizing laccase and ABTS within the MOF membrane, showing how mediator confinement can expand the oxidative range of immobilized laccase (Lou et al. 2023). Lac@Cu-ZIF-90 also improved Reactive Deep Green KE-4BD decolorization, although dye adsorption by the ZIF host may contribute to color removal (Yang et al. 2023b). MOF-based immobilization is not defined by high surface area alone, but by how the framework microenvironment regulates enzyme protection, mediator localization and dye access. The main trade-off is that the same porous or adsorptive structure that stabilises laccase and enriches dyes may also introduce diffusion resistance or adsorption-dominated color removal.

Despite their advantages in improving laccase loading, catalytic stability and dye enrichment, nanomaterial-based immobilization systems require careful evaluation of environmental safety before practical implementation. The release of free nanoparticles or nanofragments during operation, regeneration or disposal may introduce potential ecological risks, particularly for aquatic microorganisms and higher trophic organisms. Metal-containing nanomaterials and metal–organic frameworks require additional consideration because partial dissolution or metal-ion leaching may alter the toxicity profile of treated wastewater. Therefore, the long-term stability of the carrier, nanoparticle retention efficiency and possible transformation products should be systematically evaluated under realistic wastewater conditions. The environmental impact of nanomaterial-supported laccase systems depends strongly on material composition, particle size, surface modification and operational conditions. Carbon-based nanomaterials generally show high chemical stability and tunable surface properties, but their persistence and potential biological interactions require further assessment. Similarly, MOFs provide highly controllable porous structures for enzyme protection; however, framework degradation and metal release under variable pH and ionic conditions remain important concerns. Consequently, future development should prioritize environmentally responsible nanomaterials, including biodegradable or recoverable carriers, together with standardized ecotoxicological assessment, life-cycle analysis and long-term monitoring to ensure that improved catalytic performance does not introduce secondary environmental risks.

### Other nanomaterials

In addition to CNMs and metal materials, various other nanomaterials, such as biopolymers, clay minerals, and supramolecular materials, have shown considerable potential for laccase immobilization.

A representative example is the immobilization of PersiLac1 on quinoa husk-derived nanocellulose (Ariaeenejad et al. 2022). This study is particularly relevant because it links agricultural-waste valorization with enzymatic dye treatment. The nanocellulose carrier, prepared from quinoa husk, enabled an adsorption-assisted degradation process in which dye molecules were first enriched near the carrier surface and then oxidized by immobilized laccase. Compared with pristine nanocellulose, enzyme immobilization increased MG removal from 54% to 98% and CR removal from 12% to 60%, indicating that adsorption alone was insufficient and that catalytic oxidation substantially improved dye removal. The contrast between MG and CR also shows that immobilized laccase performance is strongly affected by dye charge and carrier–dye affinity, rather than being determined solely by enzyme activity. The system retained 83% MG removal after 18 cycles and was further tested in real textile effluent, giving it stronger application relevance than studies limited to single-dye model solutions. However, the lower CR removal suggests that even biomass-derived nanocarriers remain dye-selective, and that decolorization should be interpreted together with dye structure, adsorption contribution and wastewater composition.

Other carriers support the same conclusion but from different material perspectives. Chitosan/HNT beads improved laccase storage stability and enabled repeated Remazol Red RR treatment, yet efficient long-term decolorization still required mediators such as HBT or violuric acid (Hürmüzlü et al. 2021). Thiolated magnetic HNTs strengthened enzyme anchoring through disulphide linkages and allowed magnetic recovery, but their high DR80 removal was mainly demonstrated in mediator-assisted model systems (Kadam et al. 2020). LDH/alginate beads improved reusability and achieved moderate removal of MB and CV, although the system was still evaluated under controlled model-dye conditions (Nouaa et al. 2025).

Compared with conventional carbon- or metal-based nanocarriers, they provide more flexible routes to bio-derived, structurally processable and application-oriented immobilization formats. Their future relevance will therefore depend less on isolated decolorization efficiency than on whether such material formats can make laccase treatment easier to operate, recover and integrate into wastewater-processing workflows.

### Non-nanomaterials

Non-nanoscale immobilized laccase systems provide a complementary route to nanomaterial-based supports, combining high catalytic efficiency with low cost and good processability in dye decolorization.

Among macroscopic carriers, chitosan-based substrates are particularly attractive because of their high laccase loading capacity, operational stability, and biodegradability. Aslam et al. (2021) immobilized laccase on chitosan beads, achieving up to 90% decolorization of textile dye wastewater. Glutaraldehyde cross-linking enhanced stability, with 54.21% activity retention after eight cycles for Foron turquoise dye, confirming practical utility. This work exemplifies a rational enzyme-immobilization strategy in which glutaraldehyde-mediated cross-linking reinforces the structural integrity of chitosan beads and establishes stable Schiff base linkages with laccase, thereby preserving its multi-copper active sites for sustained radical-driven oxidation of diverse dye structures. More importantly, the treatment reduced BOD, COD and TOC by 92.25–96.55%, 91.90–94.94% and 77.01–93.29%, respectively. This makes the system more informative than color-removal-only examples, because it links decolorization with broader improvement in wastewater-quality indicators. Citric-acid-cross-linked chitosan–clay beads provide a glutaraldehyde-free alternative, with efficient synthetic-dye removal and retained reusability (Dos Santos et al. 2026).

The auxiliary examples clarify that non-nanoscale carriers should not be treated as equivalent supports. Chitosan/bentonite beads mainly address the mechanical weakness of simple chitosan, increasing immobilization yield from 83.6% to 88.4% and activity from 140 to 151 U/g; their key contribution is carrier reinforcement rather than a fundamental change in catalytic chemistry (Mehandia et al. 2020). By contrast, dopamine-modified sodium alginate/PEG hydrogels function primarily as protective matrices, retaining 70.23% activity after 30 days and 90.23% after six cycles, although their strongest evidence remains centerd on RB 19 under controlled conditions (Su et al. 2024). Amine-reactive PEG hydrogels prepared through thiol–yne chemistry provide another material-design route for laccase immobilization and dye degradation, extending hydrogel design beyond physical entrapment (Bat-Ozmatara and Gevrek 2026). Geopolymer microspheres further shift the design logic from enzyme retention to electron-transfer control: co-immobilization of laccase and ABTS increased CR degradation to 94.78%, but this improvement remained mediator-dependent (Shan et al. 2022).

Taken together, immobilization reframes laccase-mediated dye decolorization from a soluble-enzyme reaction into a recoverable catalytic format. Accordingly, Table 4 summarizes immobilized laccase systems using a standardized set of criteria, including enzyme source, carrier type, immobilization strategy, dye substrate, operating conditions, decolorization efficiency, reusability, mediator dependence and evidence for chemical transformation. This format highlights the distinction between systems that achieve rapid color removal through adsorption effects and those demonstrating true enzymatic degradation supported by analytical verification. Across nano- and non-nanoscale supports, meaningful comparison should extend beyond peak color removal to enzyme loading, retained activity, leakage, mediator dependence, mass transfer and reuse stability. However, most systems are still evaluated under model or simulated conditions, and their performance should therefore be interpreted as carrier-level feasibility rather than evidence of process maturity. From an engineering perspective, immobilized laccase systems occupy an intermediate position between conventional biological treatment and physicochemical processes. Unlike adsorption technologies, immobilized enzymes can potentially achieve pollutant transformation rather than simple transfer of contaminants to another phase. Compared with membrane-based separation, they avoid some fouling-related limitations but may suffer from gradual activity loss. Compared with chemical oxidation, they require less harsh operating conditions, while generally provide lower oxidation capacity. Therefore, hybrid systems combining enzymatic treatment with adsorption, membrane filtration or advanced oxidation may represent a more realistic pathway for industrial wastewater treatment. For practical implementation, immobilized laccase systems must also be evaluated under continuous reactor configurations rather than only repeated batch cycles. Packed-bed, membrane and fluidized-bed reactors may improve catalyst retention and reduce enzyme recovery costs, but they introduce additional challenges including pressure drop, channeling, carrier fouling and gradual activity loss. Thus, the suitability of an immobilization material for industrial application depends not only on its initial decolorization efficiency, but also on its ability to maintain catalytic performance during prolonged continuous operation. Consequently, the performance of immobilized laccases in dye degradation cannot be evaluated solely by decolorization percentage in batch experiments. Parameters, such as retained catalytic activity, substrate diffusion, enzyme leakage, dye transformation products, toxicity reduction, and long-term operational stability are essential indicators for determining whether immobilized systems can advance toward practical wastewater treatment.

**Table 4:** Comparison of immobilized laccase systems for dye decolorization

Immobilization method	Carrier	Laccase source	Dye	Decolorization performance	Operational performance	References
Adsorption	SP/ (PEI/PAA)_4.5_ and SP/(PEI/PMANa)_4.5_	*T. versicolor*	IC	Under 50 mg/L IC with SA, complete discoloration was achieved within 45 min.	Completely discolor the dye sample up to 4 reuse cycles	Petrila et al. (2025)
Plasma activation and GA cross-linking	(PEPP) nonwoven	*Aspergillus oryzae*	Acid Blue 74 Acid Blue 62	Decolorization rate of 85% and 42%	Retained 60% activity after 6 washes and 40% after 10 reuse cycles	Song et al. (2025)
In situ self-assembly and MOF encapsulation	g-C_3_N_4_/BC-based photo-enzyme system (BCPC)	–	MB	Under 20 mg/L dye and visible light for 10 min, MB degradation reached 100%	Retained a degradation efficiency of over 75% for MB after 10 reuse cycles	Yu et al. (2024)
Covalent, electrostatic or supramolecular immobilization	Fe_3_O_4_@SiO_2_	*T. versicolor*	MB	Decolorization rate of 47.6%	Immobilization yield 21.7–53.5%; >88% activity retained after 13 magnetic reuse cycles	Oraby et al. (2025)
Magnetic retention in polymeric filters	Magnetite	*Pycnoporus sanguineus* CS43	CR	The laccase-containing filter achieved a decolorization rate of 27%	Reached its half-life after seven cycles	Sotelo et al. (2022)
Covalent binding	Fe_3_O_4_@C	–	MG BG CV	Under 60 min photothermal treatment, MG, BG, and CV removal reached 99.8%, 99.9%, and 96.4%, respectively	Activity recovery 118.2%; after 10 batches, removal remained 99.3%, 97.4%, 94.0% and 95.1%, respectively	Wei et al. (2024)
One-pot encapsulation	Cu (PABA)	*T. versicolor*	Direct Red 31	Achieved a decolorization rate of 92% within 6 h at pH 4.5 and 40 °C	70% removal retained after 10 cycles	Jiang et al. (2022)
GA-mediated covalent immobilization	FS@cubes-PDA@PVDF	–	CR	Decolorization rate of 97.1% at pH 7.0 and 35 °C	75% retained after five reuse cycles	Zhu et al. (2020)
Entrapment / cross-linking	Dopamine-alginate beads with yeast cell walls	*Streptomyces cyaneus*	RB 5 EB	Under pH 5, room temperature and 48 h, dyes were completely decolorized without mediator	After 10 cycles, decolorization retained 90% for EB and 33% for RB 5	Popović et al. (2021)
One-step co-precipitation / encapsulation	CoCu-MOF	–	CR	Reached 90% at pH 4.0 for 1 h	Enzyme-carrying capacity and activity were 2-fold and 3.5-fold higher than Lac@Cu-MOF; >75% removal retained after six cycles	Li et al. (2022b)
Glutaraldehyde cross-linking	Amino-functionalized Cu-MOF	–	CR	Achieved a decolorization rate of 95% at pH 3.5 and 50 °C	Retained 84.63% after six reuse cycles	Li et al. (2023)
Covalent grafting	CoCu-MOF-OH-APTES	–	CR	> 95% CR removal within 1 h at pH 3.5 and 50 °C	Retained 89.18% after six reuse cycles; Relative enzyme activity reached 264.02% after six stability cycles	Long et al. (2023)
Encapsulation or surface adsorption	CuF-MOF	*Thermus thermophiles* SG0.5JP17-16	CR	Decolorization rate reached 90% after 6 h	–	Sun et al. (2024)
Adsorption immobilization	BNC	*Coriolus versicolor*	RB 19	Decolorization rate reached 88% at 55 °C and pH 4.0 after 5 h	Improved oxygen availability, reusability, COD/TOC removal and detoxification	Yuan et al. (2020)
Physical adsorption	LMS-DEGDMA	*Myceliophthora thermophila*	Lanaset^®^ Violet B	Under 20 mg/L, pH 5.0 and 37 °C, Lanaset^®^ Violet B decolorization reached 90% within 24 h	Achieved a decolorization rate of 90% and retained 63% after seven reuse cycles	Salih et al. (2023)
Adsorption	TiO_2_/ZnO/LTV	*T. versicolor*	RB5 Acid Green 25	Decolorization rate of 99% and 70%	Activity retained after 10 cycles	Kołodziejczak-Radzimska et al. (2021)
Enzyme–nanozyme hybrid immobilization / membrane casting	Fe-SS@PEG/PVDF	–	IC	> 99% IC removal in aqueous solution within 20 min	Lac@Fe-SS retained 80% activity after five cycles	An et al. (2025)
Glutaraldehyde covalent binding	Fe_3_O_4_@SiO_2_	Crude laccase	MG BG RB 19 Azophloxine	Decolorization rates of 99.6%, 99.5%, 94.6% and 94.2% under 30–60 min of illumination	Loading 10.5 mg/g; activity recovery 109.7%; after 10 cycles, removal remained 98.7%, 99.3%, 88.8%, 79.0%, 78.7% and 64.4%; leakage	Zhu et al. (2022)
Physical immobilization	Casein@CoFe_2_O_4_	*T. versicolor*	CV	Decolorization rate of 96%	50% activity retained after 24 reuse cycles; LC–MS identified less toxic metabolites	Hariri et al. (2022)
Adsorption	MOF-808	*Trametes trogii*	Reactive Blue 198 Reactive Blue 171	Decolorization rate of 77.2%, 56% after eight reuse cycles	Reused seven times without > 50% residual-activity loss; storage activity declined to 30% after 4 weeks at 25 °C	Bakar et al. (2024)
Encapsulation plus mediator co-immobilization	Cu/ZIFs MOF	*T. versicolor*	MG	Achieved a degradation rate of 100% within 4 h	Retained over 69.4% degradation efficiency after five reuse cycles	Yang et al. 2024)
Pressure-assisted self-assembly / electrostatic immobilization	SCOF/UiO-66-NH_2_	–	MG	Decolorization rate of > 98%	–	Wang et al. (2025b)
Encapsulation	MOF-PVA/CMC	–	CR	Achieved a decolorization rate of 96.2% after 5 h at pH 4 and 40 °C	Retained 55.8% activity after 10 reuse cycles; CR decolorization remained > 80% after 8 cycles; 65.8% activity after 30 d storage.	Wang et al. (2025a)
Vault encapsulation	Vault nanopartifcles	*T. versicolor*	RB 19 AO 7	Under 50 mg/L dye, 100 U/L enzyme and 27 °C, RB19 decolorization reached 72%	–	Gao et al. (2022)
Cross-linked enzyme aggregates	CLEAs	*T. versicolor* IBL-04	CV RB 5	Decolorization rate of 90.63% and 78.72%	> 60% degradation efficiency retained after 8 uses	Bilal et al. (2021)
Immobilization on cross-linked hydrogel	chitosan hydrogel	*Aspergillus* spp.	MB	–	Retained a decolorization rate of 75% after 12 reuse cycles	Sannino et al. (2023)
In situ HOF encapsulation and hydrogel construction	CMC/PVA/Lac@HOF-101 hydrogel	*T. versicolor*	AO 7	Decolorization rate of 82.5% for 5 h	66.7% activity retained after 5 cycles; ~60% removal was maintained in the presence of metal cations	Yang et al. (2023a)
Adsorptive immobilization on cross-linked hydrogel	NH_2_-terephthalic acid crosslinked chitosan-bentonite composite	*T. versicolor*	CR Brilliant Blue	Degraded > 90% CR and > 75% Brilliant Blue.	–	Ain et al. (2024)

## Application of laccase in actual wastewater

The performance of laccase systems in real wastewater is critical for evaluating their environmental relevance, because real textile effluents are far more complex than single-dye model solutions. In addition to dyes, textile wastewater often contains salts, surfactants, sizing agents, suspended solids, residual oxidants or reducing agents, and other auxiliary chemicals. These components may inhibit laccase activity, interfere with mediator cycling, increase mass-transfer resistance, or foul immobilized carriers and membranes. Therefore, high decolorization efficiency in model dye solutions should be considered preliminary evidence, whereas real wastewater treatment and continuous operation provide stronger support for process applicability. However, even successful laccase-based treatment of real textile wastewater may not always achieve complete compliance with discharge regulations when applied as a standalone process. Laccase primarily targets chromophoric and oxidizable organic structures, whereas residual dissolved organic matter, inorganic salts, nitrogen-containing compounds, metals and low-molecular-weight oxidation products may remain after enzymatic treatment. Consequently, additional treatment steps such as biological polishing, adsorption, membrane filtration, advanced oxidation or physicochemical processes may still be required depending on wastewater characteristics and regulatory limits.

Among engineered laccases, the *Cyathus bulleri* variant LCC1-62 (I490M) reported by Dahiya et al. (2023) is a representative example linking enzyme engineering with real-wastewater treatment. Compared with the WT, LCC1-62 showed more than 50% higher decolorization efficiency in real textile wastewater without mediators. In an enzyme membrane reactor, with fresh laccase supplied once the activity decreased to approximately 50 U/L, the system operated for more than 80 h, maintaining approximately 95% color removal and achieving 96% mutagenicity reduction. This example is stronger than conventional batch assays because it combines real effluent, mediator-free treatment, reactor operation and toxicity-related evaluation. However, the enzyme-replenishment strategy also indicates that activity loss remained an operational issue, making enzyme production yield, replacement frequency and long-term reactor stability important for further scale-up. Despite these advantages, such reactor demonstrations should not be interpreted as evidence that laccase alone can universally replace complete wastewater treatment trains. Regulatory compliance requires simultaneous control of multiple parameters, and enzymatic oxidation may generate intermediate compounds that require further biological degradation or adsorption-based removal. Therefore, the most realistic industrial strategy may involve integrating laccase reactors as selective pretreatment or polishing units within combined treatment systems rather than using them as isolated treatment technologies.

Immobilization provides a route to improve laccase recovery and repeated use, but real wastewater also exposes carrier-dependent limitations that are less apparent in model dye solutions (Al-Sakkaf et al. 2023). For example, PersiLac1 immobilized on Co-doped NiO nanoparticles achieved over 95% decolorization of high-strength CR textile wastewater and retained 87.66% of its initial activity after 20 cycles, indicating that immobilization can improve operational persistence under complex matrices (Ariaeenejad et al. 2023). Similar trends have been reported for laccase@BiPO_4_ hybrid nanorods and pine needle biochar-supported laccase, suggesting that carrier composition influences activity retention, matrix tolerance and reusability (Pandey et al. 2023; Tabib et al. 2024). However, these advantages must be balanced against carrier recovery, pore blockage, surface fouling, nanoparticle release and, for metal-containing supports, possible leaching. Reactor-based configurations further shift the evaluation from catalytic activity to process stability. A magnetic ring microreactor containing immobilized laccase achieved only moderate removal of BOD, ammonia and total Kjeldahl nitrogen from real wastewater, whereas a clay–chitosan filled-bed reactor with co-immobilized laccase and AS achieved 78% decolorization together with 87% COD and 91% BOD removal from real textile wastewater during 120 h of uninterrupted operation (Peñaranda et al. 2022; Mehandia et al. 2022). This contrast shows that immobilized laccase systems should not be assessed solely by peak color removal; in real dye wastewater, their relevance depends on whether dye decolorization can be sustained together with carrier integrity, water-quality improvement and manageable losses in activity, mediator and mass transfer.

Laccase systems have progressed from model-dye decolorization toward more application-relevant configurations. Nevertheless, their further translation still depends on long-term operational stability, enzyme and mediator economy, carrier durability, and more consistent evaluation of COD/TOC removal, toxicity reduction and wastewater-matrix effects. Continuous and pilot-scale reactor studies remain relatively limited compared with the large number of batch-scale investigations. Existing reactor demonstrations indicate that laccase systems can achieve sustained dye removal when enzyme retention, catalyst recovery and wastewater flow conditions are appropriately controlled; however, long-term operation often reveals challenges that are not apparent in batch experiments. These include progressive enzyme deactivation, carrier deterioration, fouling by wastewater components, oxygen transfer limitations and fluctuations in pollutant composition. Future pilot-scale studies should therefore integrate realistic hydraulic conditions, continuous monitoring of enzyme activity, catalyst lifetime evaluation and comprehensive assessment of treatment performance beyond color removal, including COD reduction, toxicity attenuation and operational cost. Such studies are essential for bridging the gap between laboratory-scale innovation and industrial wastewater treatment applications.

## Current challenges and future perspectives

During the last five years, research on laccase-based dye treatment has gradually shifted from enzyme discovery toward integrated catalyst development. Recent studies have increasingly combined novel enzyme mining, recombinant production, molecular engineering and immobilization technologies to address limitations associated with catalytic efficiency, environmental stability and operational recovery. Metagenomic approaches have enabled the identification of novel laccases with improved tolerance to challenging wastewater conditions and enhanced dye-decolorization potential, while recombinant expression systems have promoted the production and characterization of promising enzymes from diverse microbial sources (Lima et al. 2022; Ariaeenejad et al. 2023, 2024; Muhammad et al. 2024; Huang et al. 2024). In parallel, molecular engineering strategies, including rational modification, directed evolution and site-specific mutagenesis, have been applied to improve catalytic activity, stability and substrate specificity of laccases for dye degradation applications (Barber-Zucker et al. 2022; Trubitsina et al. 2024; Li et al. 2025b). Advanced immobilization platforms based on metal–organic frameworks, graphene-derived materials, biochar, hydrogels and magnetic nanostructures have further improved enzyme recyclability, operational stability and practical applicability in wastewater treatment systems (Alvarado-Ramírez et al. 2024; Chen et al. 2025; Wang et al. 2025a). Nevertheless, despite these advances, most recent reports still evaluate laccase systems under controlled laboratory conditions, and systematic studies involving mixed industrial wastewater, pilot-scale reactors and long-term operation remain limited (Brugnari et al. 2021; Dahiya et al. 2023). Therefore, further efforts are required to bridge the gap between laboratory-scale enzyme development and scalable laccase-based wastewater treatment technologies.

**Fig. 11:**
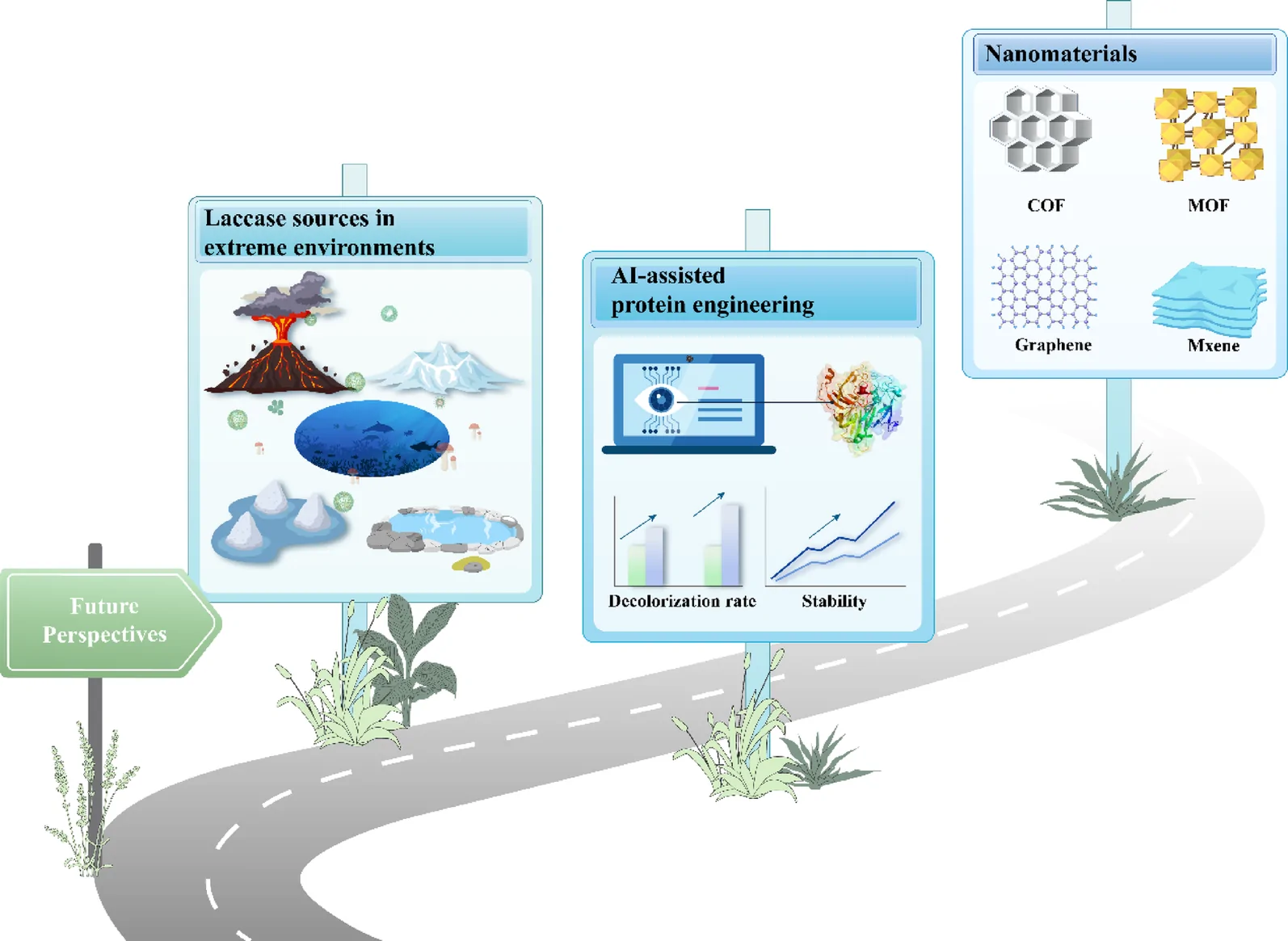
The future prospects of laccase-based dye

Although laccase-based dye decolorization has advanced substantially, its translation from laboratory studies to industrial wastewater treatment remains constrained by several unresolved challenges (Fig. 11). These limitations involve enzyme stability under complex wastewater conditions, restricted oxidation capability toward structurally diverse dyes, mediator dependence, economic feasibility, limitations associated with catalyst immobilization, and insufficient validation in continuous and large-scale treatment systems. Because these barriers arise from distinct aspects of laccase catalysis, biocatalyst development, and process engineering, they should be considered separately before proposing future solutions. A major challenge is maintaining laccase activity under realistic wastewater conditions. Although several reported laccases exhibit excellent decolorization efficiency toward individual dyes under optimized laboratory conditions, industrial textile wastewater contains high concentrations of salts, surfactants, heavy metals, fluctuating pH values, and multiple dye components that may inhibit enzyme activity or accelerate catalyst deactivation. In addition, fungal laccases with high redox potential frequently exhibit limited thermal or alkaline stability, whereas bacterial laccases generally show greater tolerance to adverse environmental conditions but may have lower oxidation capability toward high-redox-potential dyes. Achieving an appropriate balance between catalytic efficiency and environmental robustness therefore remains a fundamental challenge. Protein engineering and directed evolution provide promising approaches for improving activity and stability; however, the practical value of these strategies depends on whether enhanced catalytic performance can be achieved while maintaining economical enzyme production, purification, and replacement during long-term operation. A second major challenge is the restricted substrate spectrum of many laccases. Bulky, non-phenolic, and high-redox-potential dyes may not be efficiently oxidized through direct electron transfer and therefore often require mediator-assisted systems. Although mediators can extend the oxidative range of laccases, their application introduces additional limitations, including mediator cost, toxicity, recovery difficulty, operational instability, and the possible generation of secondary oxidation products. Consequently, the development of laccases with broader substrate specificity and higher intrinsic oxidation capacity, together with mediator-free or low-mediator-dependent systems, remains an important requirement for sustainable dye wastewater treatment. This issue is particularly important for authentic textile effluents, which contain structurally diverse dye mixtures rather than a single target compound. Economic feasibility represents another fundamental barrier to industrial implementation. Although protein engineering can substantially improve catalytic properties, industrial production of engineered laccases requires efficient expression hosts, high-yield fermentation, and cost-effective downstream purification. Improvements in catalytic efficiency achieved under laboratory conditions may not offset the costs associated with enzyme production, immobilization, regeneration, and replacement during continuous operation. Thus, future research should evaluate engineered laccases not only in terms of specific activity and decolorization efficiency but also with respect to production yield, operational lifetime, regeneration requirements, and overall treatment cost. The transition from laboratory-scale immobilized laccase systems to practical treatment processes constitutes an additional challenge. Although immobilization can improve enzyme recovery and reuse, carrier-related limitations, including enzyme leakage, pore blockage, diffusion resistance, mechanical instability, nanoparticle release, and material cost, may reduce long-term feasibility. Furthermore, many immobilized systems have been evaluated using single-dye solutions under controlled pH conditions and relatively short batch experiments rather than authentic textile wastewater. Under realistic conditions, complex mixtures of dyes, salts, surfactants, heavy metals, and other additives may inhibit enzyme activity, interfere with mass transfer, block carrier pores, and accelerate catalyst deactivation. Large-scale implementation therefore requires carriers that are inexpensive, mechanically stable, environmentally safe, resistant to enzyme leakage and fouling, and capable of maintaining effective mass transfer during prolonged operation. Reactor integration represents another critical challenge. Most engineered laccases and immobilized catalysts have been evaluated in laboratory-scale batch systems, whereas industrial application requires continuous reactors capable of maintaining stable catalytic activity over extended operational periods. Parameters such as enzyme loading, hydraulic retention time, substrate and oxygen availability, mass-transfer limitations, reactor fouling, and catalyst regeneration must be optimized simultaneously. Consequently, high decolorization efficiency in a batch experiment cannot by itself establish the suitability of a laccase system for continuous treatment, where catalyst aging, diffusion limitations, fluctuations in wastewater composition, and progressive activity loss may substantially influence performance. Insufficient process-level validation remains a major barrier to commercialization. Many studies report decolorization of synthetic dyes rather than authentic industrial textile wastewater, and the use of optimized single-dye systems may not accurately predict performance in complex effluents. Practical implementation requires pilot- and large-scale demonstrations, continuous operation over extended periods, stable catalyst performance, and systematic evaluation of treatment capacity, energy consumption, chemical requirements, operating and replacement costs, and compliance with wastewater discharge requirements. Techno-economic assessment and cost–benefit analysis are therefore essential to determine whether improvements achieved through enzyme engineering, mediator reduction, or immobilization can translate into economically competitive treatment processes. Environmental and regulatory considerations must also be incorporated into the development of next-generation laccase technologies. The use of synthetic mediators, metal-containing nanomaterials, and newly engineered enzymes requires careful assessment of toxicity, biodegradability, catalyst release, persistence, and potential ecological impacts. Moreover, effective color removal does not necessarily indicate complete detoxification, because oxidation intermediates or transformation products may retain biological activity or environmental relevance. Future research should therefore move beyond maximizing dye decolorization efficiency and prioritize sustainable enzyme production, broad-substrate and environmentally robust laccases, reduced mediator dependence, safe and recyclable immobilization materials, low-cost catalyst recovery, and stable long-term reactor performance. Comprehensive life-cycle assessment, techno-economic analysis, environmental risk assessment, and pilot-scale validation will be essential for determining the realistic industrial potential of next-generation laccase-based dye wastewater treatment systems.

Matrix tolerance is a prerequisite for treating real dye wastewater. Textile effluents contain salts, surfactants, heavy metals, auxiliary chemicals, mixed dyes and fluctuating pH, all of which can reduce laccase activity and shorten operational lifetime. Laccases from hydrothermal vents, deep-sea sediments, saline–alkaline lakes, volcanic regions and other extreme habitats may provide naturally stable scaffolds with improved resistance to heat, salt or pH stress. Their future value, however, should be assessed under wastewater-relevant conditions, including mixed dyes, high salinity, surfactants and repeated operation, rather than only by standard activity assays. Catalytic adaptability is equally important. Many laccases show limited efficiency toward non-phenolic or high-redox-potential dyes, which often increases reliance on mediators. Although mediators expand substrate range, their cost, recovery and possible toxicity restrict broader application.

Computational approaches are expected to become increasingly important for accelerating future laccase engineering. AlphaFold and related structure-prediction platforms can provide reliable three-dimensional models of newly identified laccases, including enzymes from metagenomic and uncultured microbial sources, thereby enabling structure-guided analysis of catalytic pockets, substrate-access channels and flexible regions involved in stability. These predicted structures can serve as starting points for identifying mutation targets before experimental validation. Machine learning approaches can further establish sequence–structure–function relationships by integrating available laccase databases containing amino acid sequences, structural information and experimentally measured catalytic properties. Such models may predict mutations associated with improved redox potential, substrate affinity, thermal stability or resistance to inhibitors present in industrial wastewater. In particular, machine learning-assisted screening could reduce the number of variants requiring experimental testing during directed evolution campaigns. Molecular dynamics (MD) simulations provide complementary information by evaluating the effects of mutations on protein flexibility, conformational stability, substrate binding and electron-transfer pathways under simulated reaction conditions. For laccases, MD analysis may help explain why specific substitutions improve dye oxidation or alter mediator dependence by revealing changes in active-site accessibility and dynamic interactions between the enzyme and dye molecules. Beyond mutation prediction, computational protein design platforms offer the possibility of designing laccase variants with tailored catalytic properties, including expanded substrate specificity, enhanced stability under alkaline or high-salt conditions, and reduced dependence on synthetic mediators. However, computational predictions remain dependent on the quality of training datasets and require experimental verification, particularly under realistic wastewater conditions containing mixed dyes and inhibitory compounds. Future integration of AlphaFold-based structural prediction, machine learning, molecular simulations and high-throughput experimental screening may establish a more efficient design–build–test–learn cycle for developing industrially applicable laccases.

AI-assisted protein engineering represents an emerging direction for overcoming the limitations of conventional laccase optimization strategies. Machine learning models can analyze relationships between amino acid sequences, structural features and experimentally measured enzyme properties, enabling prediction of mutations associated with improved catalytic activity, stability or substrate adaptability. Structure-prediction approaches, including AlphaFold-based modelling, provide high-confidence structural information that can support identification of substrate-access channels, flexible regions and residues involved in electron transfer. In combination with molecular docking and molecular dynamics simulations, AI-assisted workflows can predict enzyme–dye interactions and prioritize mutations that improve compatibility with structurally diverse dye molecules. Furthermore, protein language models trained on large sequence databases may enable the discovery of previously unexplored laccase variants from environmental metagenomes and extreme habitats. However, AI-guided enzyme engineering still faces several challenges. The accuracy of prediction models depends strongly on the quantity and quality of available sequence–function datasets, while many reported laccase variants lack standardized measurements under comparable conditions. Predicted improvements must therefore be experimentally validated, particularly under mixed-dye systems and real wastewater conditions. Future progress will likely depend on integrating AI prediction with high-throughput screening, directed evolution and automated experimental platforms to establish closed-loop design–build–test–learn workflows for industrially relevant laccases. This strategy will become more powerful as sequence–function datasets and experimental validation pipelines improve. Operational recovery remains another major barrier. Immobilization can improve enzyme reuse and stability, while COFs, MOFs, graphene, MXene and related nanomaterials provide tunable surfaces for enzyme loading, dye adsorption and interfacial electron transfer. Future nanomaterial-based systems should further optimise pore structure, enzyme–carrier interactions, anti-fouling surfaces, carrier recoverability and material safety to maintain these advantages in real effluents.

To overcome enzyme instability, future studies should focus on discovering and engineering laccases from extreme environments, including saline lakes, hot springs, deep-sea environments and alkaline habitats. These enzymes may provide naturally evolved tolerance toward temperature, salinity and pH stress. However, their potential should be evaluated using mixed dyes, real textile wastewater and long-term operation rather than only conventional biochemical assays. For improving substrate specificity and catalytic efficiency, artificial intelligence-assisted protein engineering represents a promising strategy. Machine learning models, AlphaFold-based structural prediction, molecular docking and molecular dynamics simulations can identify key residues involved in substrate binding, electron transfer and structural stability. Integrating computational prediction with experimental directed evolution may accelerate the development of laccase variants specifically optimized for industrial dye degradation. Future immobilization research should move from developing carriers with high enzyme loading toward designing functional carrier materials with controlled microenvironments. Promising strategies include magnetic recyclable supports, porous frameworks, anti-fouling surfaces and bio-based carriers that simultaneously enhance dye enrichment, enzyme stability, mass transfer and catalyst recovery. Importantly, new materials should also be evaluated for environmental safety, production cost and scalability.

## Conclusion

This review summarizes recent advances in laccase-based dye decolorization and degradation from catalytic mechanisms to engineering applications. The available evidence shows that laccase performance is jointly determined by dye structure, enzyme source, mediator involvement, reaction matrix and immobilization design. Azo, anthraquinone and triphenylmethane dyes exhibit distinct degradation behaviors because of differences in chromophore structure, redox properties, steric accessibility and intermediate formation. Progress in novel laccase discovery, recombinant expression, protein engineering and immobilization has expanded the catalytic potential and operational stability of laccase systems. Fungal laccases generally provide strong oxidative capability, whereas bacterial and extremotolerant laccases offer advantages in environmental robustness. Engineered variants and immobilized biocatalysts further improve activity, tolerance, recovery and reuse, providing important routes for adapting laccases to complex wastewater conditions. Future advances in AI-assisted protein engineering may further accelerate this transition by enabling more predictive design of laccase variants with tailored catalytic properties. Nevertheless, AI-generated candidates will require systematic experimental validation under realistic wastewater conditions to confirm their practical value. Overall, the development of laccase-based dye treatment is moving beyond simple decolorization toward a more integrated stage in which catalytic mechanism, enzyme engineering, immobilization design and wastewater-process validation are considered together.

## Data Availability

No datasets were generated or analysed during the current study.
